# IGF2BP1 Promotes Proliferation of Neuroendocrine Neoplasms by Post-Transcriptional Enhancement of EZH2

**DOI:** 10.3390/cancers14092121

**Published:** 2022-04-24

**Authors:** Florian Sperling, Danny Misiak, Stefan Hüttelmaier, Patrick Michl, Heidi Griesmann

**Affiliations:** 1 Department of Internal Medicine I, Martin Luther University Halle-Wittenberg, 06120 Halle, Germany; f_sperling@yahoo.de (F.S.); heidi.griesmann@uk-halle.de (H.G.); 2 Department for Molecular Cell Biology, Institute for Molecular Medicine, Martin Luther University Halle-Wittenberg, Charles Tanford Protein Center, 06120 Halle, Germany; danny.misiak@medizin.uni-halle.de (D.M.); stefan.huettelmaier@medizin.uni-halle.de (S.H.)

**Keywords:** NEN, RNA-binding protein, IGF2BP1, EZH2, H3K27me3, cell cycle

## Abstract

**Simple Summary:**

Neuroendocrine neoplasms (NEN) are very heterogeneous malignancies arising at different sites of the body that show an increasing incidence in recent decades. Here, we show that IGF2 mRNA binding protein 1 (IGF2BP1) is highly expressed in NEN cell lines, leading to enhanced cell proliferation. This oncogenic function relies on post-transcriptional stimulation of EZH2 expression by IGF2BP1, resulting in epigenetic silencing of cell cycle inhibitors via tri-methylation of histone H3 at lysine 27 (H3K27me3). Combinatorial pharmacological targeting of IGF2BP1, EZH2, and the EZH2-activator Myc leads to synergistic antiproliferative and proapoptotic effects in NEN cells, representing a novel therapeutic strategy in neuroendocrine malignancies.

**Abstract:**

Neuroendocrine neoplasms (NENs) represent a heterogenous class of highly vascularized neoplasms that are increasing in prevalence and are predominantly diagnosed at a metastatic state. The molecular mechanisms leading to tumor initiation, metastasis, and chemoresistance are still under investigation. Hence, identification of novel therapeutic targets is of great interest. Here, we demonstrate that the RNA-binding Protein IGF2BP1 is a post-transcriptional regulator of components of the Polycomb repressive complex 2 (PRC2), an epigenic modifier affecting transcriptional regulation and proliferation: Comprehensive in silico analyses along with in vitro experiments showed that IGF2BP1 promotes neuroendocrine tumor cell proliferation by stabilizing the mRNA of Enhancer of Zeste 2 (EZH2), the catalytic subunit of PRC2, which represses gene expression by tri-methylation of histone H3 at lysine 27 (H3K27me3). The IGF2BP1-driven stabilization and protection of EZH2 mRNA is m6A-dependent and enhances EZH2 protein levels which stimulates cell cycle progression by silencing cell cycle arrest genes through enhanced H3K27 tri-methylation. Therapeutic inhibition of IGF2BP1 destabilizes EZH2 mRNA and results in a reduced cell proliferation, paralleled by an increase in G1 and sub-G1 phases. Combined targeting of IGF2BP1, EZH2, and Myc, a transcriptional activator of EZH2 and well-known target of IGF2BP1 cooperatively induces tumor cell apoptosis. Our data identify IGF2BP1 as an important driver of tumor progression in NEN, and indicate that disruption of the IGF2BP1-Myc-EZH2 axis represents a promising approach for targeted therapy of neuroendocrine neoplasms.

## 1. Introduction

Neuroendocrine neoplasms (NENs) are heterogenic malignancies with rising incidence [1]. NEN originate from all sites of the neuroendocrine system, being most prevalent in the gastrointestinal tract and the lung [2]. NENs can generally be classified into low (G1) and intermediate (G2) tumors, as well as high-grade (G3) carcinomas according to their proliferation rate [3]. Low grade tumors are the more frequent subgroup with slow disease progression and long survival rates. The subset of aggressive, high-grade carcinomas is usually associated with poor outcomes [4]. The diagnosis of NENs remains challenging, as symptoms may occur only late in metastatic stages of the disease [5]. If curative surgery is not feasible, there is an indication for locoregional or systemic therapies including targeted therapies or chemotherapy [6]. In addition to temporary positive effects on tumor growth control, patients frequently develop resistance to systemic therapies, emphasizing the clinical need for novel targeted approaches [7].

Most NENs occur sporadically, but there are also hereditary predispositions, such as multiple endocrine neoplasia type-I (MEN-1) or -II (MEN-2) [8]. In sporadic NEN, whole genome sequencing revealed somatic mutations in MEN1 and mTOR pathway genes [9]. Additionally recent analyses identified mutations in DNA repair genes (MUTYH, CHEK2, BRCA2) and in genes associated with chromatin remodeling, DNA damage repair and telomere maintenance [10]. Epigenetic alterations during NEN development, potentially leading to new insights in tumor formation have gained special interest [11,12,13]. Nevertheless, the molecular pathogenesis of NENs remains to be further investigated and identification of biomarkers for prognosis and targeted treatment options remains an ongoing challenge. 

Recently, we have identified the RNA binding protein IGF2BP1 as a transcriptional super-enhancer of E2F-driven gene expression promoting an enhanced G1/S cell cycle transition in diverse solid cancers including pancreatic ductal adenocarcinoma (PDAC) [14].

IGF2BPs represent a family of RNA-binding proteins (RBP) comprised of three members (IGF2BP1–3). All three IGF2BPs show abundant expression during embryogenesis, with only IGF2BP2 remaining ubiquitously expressed in adult tissues [15]. In several studies, an enhanced expression of IGF2BP proteins could be demonstrated in various tumor entities, with IGF2BP1 and IGF2BP3 showing bona-fide oncofetal, but distinguished expression patterns [15,16]. IGF2BP proteins are generally thought to support pro-oncogenic, pro-proliferative cancer phenotypes with IGF2BP1 being the most conserved posttranscriptional regulator within the family [17]. Its tumor-cell promoting role was shown in ovarian and hepatocellular carcinomas, as well as in neuroblastomas [17,18,19]. IGF2BP1 enhances mesenchymal-like tumor cell phenotypes by promoting tumor cell migration, invasion, and metastasis [20,21]. In addition, the protein augments general oncogenic features, such as tumor cell proliferation, self-renewal, anoikis-resistance, and drug-resistance [22,23,24]. As the post-transcriptional regulator IGF2BP1 controls the transport, translation and degradation of numerous target mRNAs [15], among them MYC mRNA. IGF2BP1 has been described to protect MYC mRNA from degradation by endonucleases, thereby promoting pro-oncogenic capabilities through sustaining the expression of the transcription factor that is also relevant for NEN development and progression [25,26]. Recently it was proposed that the regulation of the MYC mRNA by IGF2BP1 relies on m6A (N6-methyladenosine) post-transcriptional modification. IGF2BP1 was mechanistically described as an “m6A-reader”, showing preferred association with m6A-modified target mRNAs, resulting in the protection of target mRNAs from decay and, consequently, elevated protein synthesis [27]. As the targeting oncogenic MYC in cancer remains challenging, alternative approaches are under investigation, for instance with the BET-inhibitor OTX015 [28,29]. Recently, the agent BTYNB, a novel small molecule inhibitor of IGF2BP1-mRNA association, has been described as a new approach to impair MYC expression [30].

Independently of MYC, numerous additional targets were identified to be regulated by IGF2BP1, including SRF, LIN28B, HMGA2, and MDR1 [14,23,30,31]. This emphasizes the broad impact which IGF2BP1 might have on tumor-promoting pathways.

So far, only limited data on IGF2BP1 exist in endocrine tissues and NENs. Interestingly, IGF2BP2 has been identified as a potential mediator of pancreatic islet proliferation and cell cycle regulation in adipose tissues of a murine model of type 2 diabetes [32]. In neuroendocrine small-cell lung cancer cell lines, enhanced IGF2BP2 expression is associated with drug resistance.

Here, we demonstrate that IGF2BP1 promotes proliferation of neuroendocrine cancer cells by regulating the histone-lysine N-methyltransferase Enhancer of Zeste homolog 2 (EZH2) at the post-transcriptional level. IGF2BP1 enhances the expression of EZH2 in an m6A-dependent manner leading to the repression of cell cycle arrest genes. Moreover, pharmacological inhibition of IGF2BP1-mediated EZH2 expression led to cell cycle arrest and apoptosis induction in neuroendocrine cancer cell lines.

## 2. Materials and Methods

### 2.1. Reagents

Actinomycin D, BTYNB, DZNep and OTX015 were purchased from Cayman Chemical. The compounds were dissolved in DMSO.

### 2.2. Cell Cultures and Growth Conditions

COLO320DM and NCIH727 cells were obtained from the ATCC (Germany) and cultured in RPMI1640 (Life Technologies, Darmstadt, Germany) with 10% fetal calf serum (FCS, Capricorn Scientific, Ebsdorfergrund, Germany). BON1 cells were a kind gift of R. Göke (University of Marburg, Germany) and cultured in DMEM/HAM’s F12 (Life Technologies, Darmstadt, Germany) with 10% FCS. All cells were cultured in a humidified atmosphere containing 5% CO_2_ at 37 °C. The absence of mycoplasma contamination in the cultured cell lines was verified by qRT-PCR prior investigations. The following primers from Drexler and Uphoff (2002) [33] were used on an ABI 7500 instrument (Thermo Scientific): forward 5′-YGC CTG RGT AGT AYR YWC GC-3′ and reverse 5′-GCG GTG TGT ACA ARM CCC GA-3′.

### 2.3. Transfection of siRNAs

Transfections of siRNAs were performed using Lipofectamine RNAiMAX (Invitrogen, Darmstadt, Germany) according to manufacturer’s instructions. Transfection of si-IGF2BP1, si-MYC, and si-METTL3/14 was performed at a final concentration of 10 nM and 20 nM for si-EZH2. siRNA sequences are listed in Table S1.

### 2.4. Cell Proliferation and Viability Assays

Cell proliferation was determined by cell counting using the Neubauer chamber and analyzed. For knockdown-experiments 1 × 104 and inhibitor-treatments 3 × 104 BON1, COLO320 or NCIH727 cells per well were initially plated in triplicates in a 48-well plate for each time point. Cells were harvested 24 h, 48 h and 72 h after seeding and absolute cell count was determined. 

Cell viability was analyzed using the CellTiter-Glo^®^ Luminescent Cell Viability Assay (Promega, Walldorf-Mannheim, Germany) according to manufacturer’s instructions. For knockdown and inhibitor-treatment experiments 5 × 103 cells were seeded in triplicates in a volume of 100 µL in a 96-well plate. Then, 72 h after seeding 100 µL of CellTiter-Glo^®^-reagent was added to every well and luminescence was measured using the Luminoskan Ascent (Thermo Scientific, Darmstadt, Germany). Results were reported as relative light units (RLU).

### 2.5. Flow Cytometry Analysis of Cell Cycle and Apoptosis

Cell cycle analysis was performed after fixation of the cells in 70% ice cold ethanol using FxCycle PI/RNase Staining Solution (Thermo Scientific, Darmstadt, Germany), according to the manufacturer’s instructions. Apoptosis was analyzed by Annexin V-FITC and Propidium iodide staining. Cells were harvested, washed, and resuspended in 1× binding buffer. Cells were incubated with Annexin V-FITC (BioLegend, Koblenz, Germany) for 20 min and Propidium iodide (Sigma-Aldrich, Darmstadt, Germany) for a further 15 min in the dark. Cells treated with UV-light for 10 min served as positive control. Flow cytometry was performed on LSRII (BD Biosciences, Heidelberg, Germany), and data were analyzed using FlowJo software (BD Biosciences, Heidelberg, Germany). 

### 2.6. RNA Sequencing and Differential Gene Expression

Total RNA from triplicates of si-IGF2BP1 and si-C transfected BON1 cells was isolated using QIAzol lysis reagent (Qiagen, Hilden, Germany) 72 h after transfection. Total RNA-sequencing library preparation and sequencing was performed on an Illumina HighScan-SQ (Illumina, San Diego, CA) at the Core Unit DNA-Technologies of the IZKF Leipzig (Faculty of Medicine, University Leipzig) using third version chemistry and flowcell according to the instructions of the manufacturer. For total transcriptome-sequencing (RNA-seq) Cutadapt (v. 1.14) was used to clip off low quality read ends and sequencing adapters. Afterwards, reads were aligned to the human genome (UCSC GRCh19) using TopHat (v 2.0.12) or Bowtie2 (V 2.2.4), respectively. For gene-mapped read summarization, FeatureCounts (v 1.4.6) was used and annotated by Ensembl (GRCh37.75). Differential gene expression (DE) expression comparing si-C to si-IGF2BP1 transfected cells was calculated by R package edgeR (v 3.12.1) using TMM normalization (Table S2). For the determination of differential expression a false discovery rate (FDR) value below 0.05 was considered as threshold.

For KEGG pathway analyses (https://www.genome.jp/kegg/pathway.html, accessed on 6 June 2018) all significantly down- or upregulated genes (FDR ≤ 0.01) were mapped to homo sapiens (hsa) genome. Functional Annotation Tool of DAVID 6.8 (https://david.ncifcrf.gov/summary.jsp, accessed on 26 June 2018) was used for analysis. The results were ranked by Bonferroni-corrected significance (Table S3).

### 2.7. Actinomycin D Treatment

For RNA stability analysis Actinomycin D (5 µg/mL) treatment was performed 24 h after siRNA transfection. Reaction was stopped by harvesting the cells and isolating RNA using Qiazol reagent (Qiagen, Hilden, Germany). DNA was digested using DNase I (Qiagen, Hilden, Germany) according to manufacturer’s instructions. RNA quality and quantity was determined by NanoDrop (Thermo Scientific, Darmstadt, Germany), reverse transcribed to cDNA and analyzed via qRT-PCR.

### 2.8. RNA Immunoprecipitation

RNA-immunoprecipitation (RIP) of BON1, COLO320, and NCIH727 cell extracts (1 × 106 per condition) was performed using the IGF2BP1 RIP-Assay Kit (MBL) following the manufacturer’s instructions. For protein-pulldown, cleared extracts were incubated with the IGF2BP1-Antibody (MBL) and Protein A/G Magnetic Beads (Biotool). The RNA was extracted according to manufacturer’s instructions including a DNA digested using DNase I. RNA quality and quantity was determined by NanoDrop (Thermo Scientific), reverse transcribed to cDNA and analyzed by qRT-PCR.

### 2.9. Quantitative Real-Time PCR (qRT-PCR) 

Total RNA of cells was isolated using the NucleoSpin RNA Kit (Macherey-Nagel, Düren, Germany) according to the manufacturer’s protocol and reverse transcribed with the Omniscript RT Kit (Qiagen, Hilden, Germany) using Oligo-dT Primers. qRT-PCR reactions were performed on an ABI 7500 Fast Realtime PCR system (Applied Biosystems, Darmstadt, Germany) using the Luna Universal SYBR Green Supermix (NEB, Frankfurt am Main, Germany) and the corresponding primers (Biomers, Ulm, Germany) listed in Table S4. The ribosomal protein RPLP0 was used as housekeeping gene. All experiments were performed in duplicates and are displayed in ±SD.

### 2.10. Western Blotting

Cell pellets were lysed in RIPA buffer (50 mM Tris-HCl (pH 7.5), 150 mM NaCl, 0.1% SDS, 1% sodium deoxycholate and 1% Triton X-100) supplemented with protease inhibitor cocktail (Complete, Roche Applied Science, Penzberg, Germany). Protein concentrations were determined with Coomassie brilliant blue (Thermo Scientific, Darmstadt, Germany). Protein samples (10–25 µg of total protein per lane) were separated by SDS-PAGE and transferred to Hybond-P polyvinylidene fluoride membranes (GE Healthcare, Boston, MA, USA). After blocking in 5% nonfat dry milk in TBS-T (0.1% Tween 20) for 1 h immunoblots were probed with primary antibodies (Table S5) over night at 4 °C followed by an incubation of peroxidase-conjugated secondary antibody (GE Healthcare, Boston, MA, USA) for 1 h according to the manufacturer’s protocol Blots were detected using WesternBright Chemiluminescence Substrate (Biozym, Hessisch Oldendorf, Germany) and the digital imaging system from Intas (Göttingen, Germany). All Western blot images have been cropped for improved clarity and conciseness. Quantification by densitometry was performed using Image J software (National Institutes of Health, Bethesda, Rockville, MD, USA). Relative band intensities were expressed as arbitrary units and normalized to the corresponding actin.

### 2.11. Transgenic Mouse Model

The RIP1-Tag2 transgenic mouse model has been described previously [34]. RIP1-Tag2 mice were purchased from NCI Mouse Repository (Bethesda, Rockville, MD, USA) and maintained in a C57BL/6N background. No glucose-enriched food or water was offered to diminish the risk of hypoglycemia. Mice were maintained in a climate-controlled specific pathogen–free (SPF) facility. All animal experiments were approved by the local government authorities and performed according to the guidelines of the animal welfare committee.

### 2.12. Statistical Analyses

Graphical data present the mean ± standard deviations (SD) of three independent experiments. All statistical analyses were performed by GraphPad Prism 8 software (San Diego, CA). Differences between groups were calculated using unpaired, two-tailed Student’s *t*-test (two groups) and one-way ANOVA with post hoc Bonferroni comparison. For all tests, *p*-values < 0.05 were considered statistically significant. *p*-Values of * *p* < 0.05, ** *p* < 0.01, or *** *p* < 0.001 are indicated in the figures.

## 3. Results

### 3.1. IGF2BP1 Is Differentially Expressed in Neuroendocrine Tumor Cells

**Figure 1:**
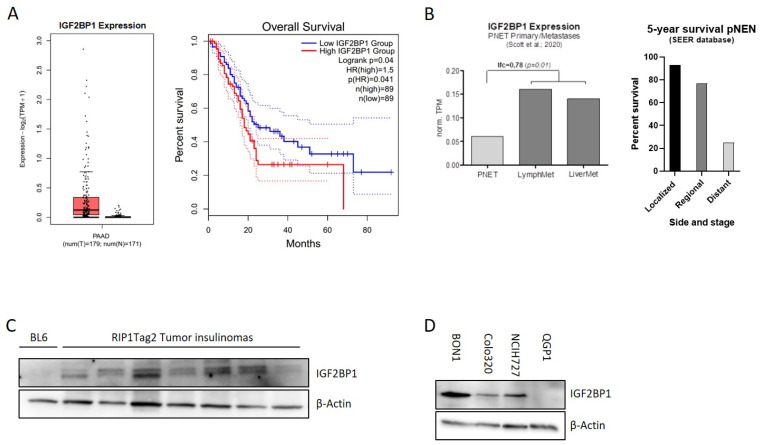
IGF2BP1 is highly expressed in pNEN and is associated with worse prognosis. (**A**) IGF2BP1 mRNA expression in PDAC tumors matched with TCGA normal and GTEx data (left panel) and median overall survival in PDAC with low (blue line) or high (red line) IGF2BP1 expression (right panel). Blot generated using GEPIA2 database. (**B**) IGF2BP1 mRNA expression in primary pancreatic neuroendocrine tumors (pNEN) compared to corresponding lymph node and liver metastasis (left panel; data derived from Scott et al., 2020), as well as 5-year survival rate of pNEN patients (right panel) depending on cancer stage: Localized (tumor diagnosed at a localized stage), regional (tumor spreading to surrounding tissues and/or regional lymph nodes), distant (tumor with distant metastases). Data from Cancer.Net. (**C**,**D**) Western blots of IGF2BP1 protein levels in pooled benign murine pancreatic Islet (BL6) and individually isolated insulinomas of RIP1-Tag2 mice (**C**), as well as whole cell lysates from BON1, Colo320 and NCIH727 NEN cell lines (**D**). Actin served as loading control. The uncropped western blot figures were presented in Figure S7.

The mRNA binding protein IGF2BP1 is highly expressed in different types of solid human cancers. Due to their heterogeneity and rareness, large-scale publicly available expression data of pancreatic NEN are missing. In contrast, IGF2BP1 expression data for pancreatic adenocarcinomas, the most frequent pancreatic tumor-type, are available indicating that IGF2BP1 expression is overall upregulated in PDAC and associated with a significantly reduced overall survival (Figure 1A). In neuroendocrine neoplasms of the pancreas (pNEN), a recently published study from Scott et al. (2020) [35] compared primary NEN samples with matched metastases and identified 902 genes differentially regulated in human pNEN metastases compared to primary tumors. Remarkably, in this study IGF2BP1 mRNA was enriched 3-fold in lymph node and liver metastases compared to the primary tumor tissue (Figure 1B), raising the hypothesis that IGF2BP1 could functionally contribute to the prometastatic phenotype leading to worsened prognosis (Figure 1B). To further investigate this hypothesis, we analyzed IGF2BP1 expression in insulinomas of 10–15 weeks old RIP1-Tag2 (RT) mice, a well-characterized pancreatic neuroendocrine tumor mouse model of ß-cell carcinogenesis [34]. IGF2BP1 protein abundance was compared to a pooled benign pancreatic islet lysate, isolated from BL6 control mice. Although IGF2BP1 was not detected in the control lysate, an expression was observed in the isolated insulinomas (Figure 1C) indicating a potential regulation and tumor-related expression in the murine neuroendocrine tumor model.

To further investigate IGF2BP1 in human neuroendocrine tumors, we examined IGF2BP1 mRNA and protein expression in human NEN cell lines derived from pancreatic (BON1, QGP1), colon (Colo320) and lung (NCIH727) primary tumors. Although high mRNA and protein levels were detected in the lymph-node metastatic cell line BON1 as well as in Colo320 and NCIH727 cell lines, the primary pNEN cell line QGP1 did not express IGF2BP1 (Figure 1D and Figure S1A).

### 3.2. IGF2BP1 Knockdown Impairs Proliferation and Viability of NEN Cells

**Figure 2:**
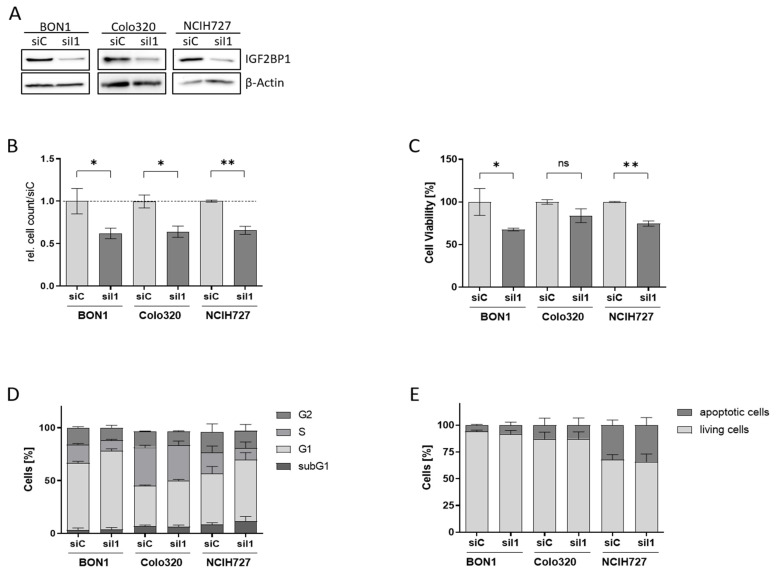
Knock-down of IGF2BP1 results in reduced proliferation and cell cycle progression in NEN cell lines. (**A**) Western blots of IGF2BP1 knockdown (siI1) in indicated NEN cell lines after 72 h. Actin served as loading control. (**B**) Cell proliferation as determined by cell counting at indicated time points upon knock-down of IGF2BP1 compared to control (siC) transfected cells. (**C**) Cell viability as determined by ATP-based CellTiter Glo assay after 72 h of IGF2BP1 knock-down in indicated NEN cell lines. Cell viability was normalized to siC transfected cells serving as control. (**D**) Cell cycle analysis of NEN cells 72 h after knockdown of IGF2BP1 showing the percentage of cells in the indicated cell cycle phases. (**E**) Apoptosis analyzed by Annexin V staining 72 h after IGF2BP1 knock-down. Annexin V and propidium iodide negative cells were summarized as living fraction and compared to positively stained apoptotic cells. Statistical significance was determined by Student’s *t*-test: * *p* < 0.05; ** *p* < 0.01. The uncropped western blot figures were presented in Figure S7.

To characterize the functional role of IGF2BP1 in NEN cells we used homolog-specific siRNA pools to knock-down IGF2BP1 expression in BON1, Colo320, and NCIH727 cells (Figure 2A, Supplementary Figure S1B). Upon IGF2BP1 knock-down a significantly reduced proliferation rate could be observed in all three cell lines (Figure 2B and Figure S1C), paralleled by a reduced cell viability compared to scrambled control cells (Figure 2C). As IGF2BP1 has been shown to promote G1/S cell cycle transition in other cancer types [14] we performed cell cycle analysis upon IGF2BP1 knockdown. As expected, we observed an increase in cells in the G1 phase accompanied by a decreased proportion of cells in the S- and G2-phase, confirming prior reports of impaired G1/S-progression upon IGF2BP1 knockdown (Figure 2D and Figure S1D). In contrast, there was no significant increase in sub-G1 population, indicating apoptosis. The absence of apoptosis upon IGF2BP1 depletion could also be corroborated by Annexin V staining (Figure 2E and Figure S2A). These data indicate that the decreased cell viability upon IGF2BP1 knockdown is a consequence of a cell cycle arrest without significant induction of apoptotic cell death.

### 3.3. IGF2BP1 Influences the RNA Landscape of NEN Cells

**Figure 3:**
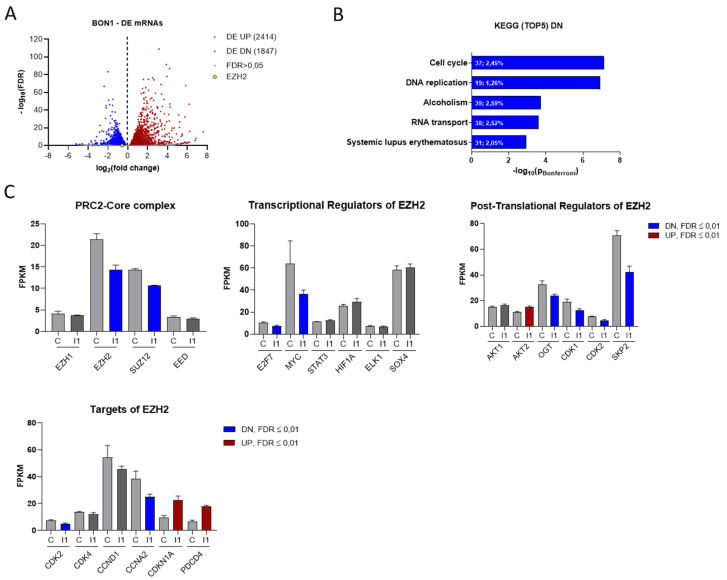
IGF2BP1 knockdown affects the transcriptional landscape of NEN cells. (**A**) Volcano plots of differentially expressed (DE) genes (threshold: FDR < 0.05) determined by RNA-seq in BON1 cells upon IGF2BP1 knockdown compared to siC transfected cells after 72 h. (**B**) KEGG- pathway analysis of mRNAs downregulated upon IGF2BP1 knockdown in BON1 cells (threshold: FDR < 0.01). Pathways are ranked by Bonferroni-corrected significance. First number indicates counts mapped to the respective pathway and second number gives percentage of these counts related to total pathway counts. (**C**) Expression of potential PRC2 components as well as EZH2 transcriptional, post-translational regulators and targets in BON1 cells after IGF2BP1 (I1) knockdown compared to siC (**C**) evaluated by RNA-seq.

To analyze transcriptional changes upon IGF2BP1 depletion, we performed RNA sequencing using BON1 cells with or without knock-down of IGF2BP1. We identified 2414 significantly up- and 1847 downregulated mRNAs (FDR ≤ 0.01) by IGF2BP1 knock-down, comprising 22.2% of all analyzed mRNAs, which emphasizes the regulatory capability of IGF2BP1 (Figure 3A). Functional clustering of differentially expressed mRNAs via KEGG-pathway analyzer (https://www.genome.jp/kegg/pathway.html, accessed on 6 June 2018) categorized most significantly downregulated mRNAs upon IGF2BP1 knock-down into ‘cell cycle’- and ‘DNA replication’- pathways (Figure 3B), consistent with the functional changes described above (Figure 2D). On the other hand, mRNAs upregulated by IGF2BP1 were associated with cell communication signaling pathways (Figure S3A). To elaborate how IGF2BP1 regulates transcriptional mechanisms, we individually analyzed the regulation of known IGF2BP1 targets and prominent cell cycle associated genes. We could confirm the well-described downregulation of MYC [25] as a target of IGF2BP1 (Figure 3C). The transcription factor c-Myc is known as one of the most important determinants of the molecular landscape of cancer cells resulting in a pro-proliferative and anti-apoptotic phenotype [36]. Surprisingly, upon IGF2BP1 knock-down we also identified decreased mRNA levels of several epigenetic modifiers of the PRC2 complex (Figure 3C), known to play an important role in the regulation of cell cycle and tumor cell proliferation [37]. In this context, we detected significantly decreased mRNA levels of EZH2 and SUZ12 (Figure 3C), two components of the PRC2 complex core subunit. As EZH2 has been described as major epigenetic regulator involved in tumorigenesis of several cancers, we further focused on its regulation by IGF2BP1. RNA-seq data revealed regulation of several known transcriptional and post-translational regulators of EZH2 upon IGF2BP1 knockdown (Figure 3C). Both the transcription factors Myc [38] and E2F7 [39], which directly bind and activate the EZH2 promoter, as well as CDK1/2 [40], SKP2 [41], and OGT [42], all of which contributes to EZH2 stability and activation, were downregulated upon IGF2BP1 knockdown. In line with these distinct alterations of epigenetic modifiers, we identified transcriptional downregulation of essential cell cycle components and EZH2 targets (Figure 3C), such as CDK2, and upregulation of well-known cell cycle inhibitors and tumor suppressors, such as CDKN1A and PDCD4 (Programmed Cell Death 4), whose expression has been associated with epigenetic regulation [43]. Subsequently, we aimed to corroborate the BON1 RNA-seq data in the additional NEN cell lines Colo320 and NCIH727 with a particular focus on the PRC2 complex. Both EZH2 and SUZ12 were significantly downregulated in all three cell lines upon IGF2BP1 knock-down (Figure S3C). In contrast, mRNA levels of EED, another component of the PRC2 core subunit, was exclusively reduced in NCIH727 cells (Figure S2C). Furthermore, decreased mRNA expression of DNMT1, a DNA methyltransferase associated with EZH2, was observed in BON1 and NCIH727 cells (Figure S3B,C). We further confirmed deregulation of MYC mRNA in BON1 and NCIH727 cells and could verify mRNA downregulation of cell cycle related components, such as CCND1, CCNE1, CDK2, as well as upregulation of their inhibitors CDKN1A and CDKN1B (Figure S3C). Moreover, upregulation of the tumor suppressor PDCD4 could be confirmed in BON1 and NCIH727 cells. Taken together, these results indicate an important regulatory impact of IGF2BP1 on the epigenetic landscape of NEN cells, thereby significantly affecting cell cycle progression and tumor cell viability.

### 3.4. EZH2 Is Regulated by IGF2BP1 in NEN Cells

**Figure 4:**
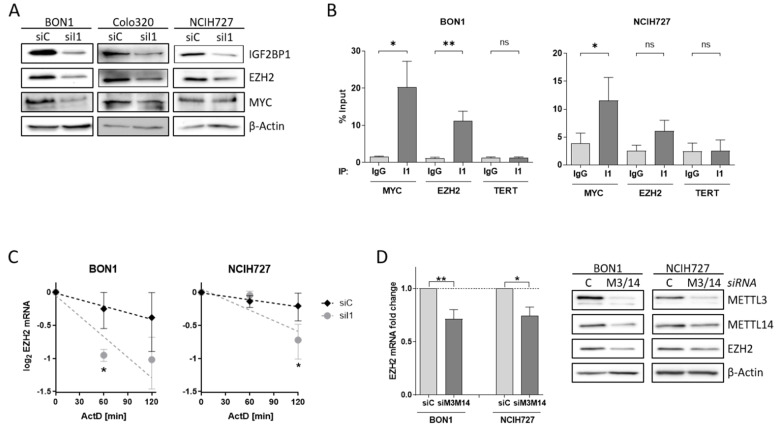
IGF2BP1 regulates EZH2 mRNA stability. (**A**) Representative Western blots of indicated proteins upon knock-down of IGF2BP1 (siI1) in NEN cells. Actin served as loading control. (**B**) RNA-Immunoprecipitation (RIP) analysis by qRT-PCR after pulldown of IGF2BP1 protein. Binding of MYC and EZH2 mRNA to IGF2BP1 was compared to IgG isotype control. TERT mRNA served as negative control and showed no accumulation. All amplifications were normalized to total RNA input. (**C**) Decay of EZH2 mRNA upon IGF2BP1 knock-down was analyzed via Actinomycin D treatment at indicated time points. (**D**) Expression of EZH2 mRNA (left panel) and protein (right panel) was analyzed upon knock-down of m6A-writers METTL3/14 after 72 h. mRNA levels were determined by qRT-PCR, normalized to ribosomal protein RPLP0 as internal standard and shown as fold change to siC. Protein expression is shown in representative Western blots. Actin served as loading. Data are representative for n = 3 independent experiments. Statistical significance was determined by Student’s *t*-test: * *p* < 0.05; ** *p* < 0.01. The uncropped western blot figures were presented in Figure S7.

To investigate the impact of alterations of the PRC2 core components in NEN cells in more detail, we analyzed the potential regulatory role of IGF2BP1 on EZH2 and SUZ12 expression. After knock-down of IGF2BP1, EZH2 protein expression was reduced in all three cell lines (Figure 4A). Previous studies have described the transcription factor c-Myc also regulating EZH2 expression in various cancers, as well as in embryonic stem cells [38,44,45,46]. Likewise, c-Myc mRNA is stabilized by IGF2BP1 in complex with other RNA binding proteins [25]. Following knock-down of IGF2BP1, c-Myc protein levels decreased in BON1 and Colo320 cells, but were not altered in NCIH727 cells (Figure 4A). The consistent regulation of EZH2 but inconsistent regulation of c-Myc upon IGF2BP1 knock-down in NEN cell lines raises the possibility that EZH2 expression may also be regulated by direct interaction with IGF2BP1 independently of c-Myc. To further investigate the regulation of EZH2 by IGF2BP1 in NEN cells we analyzed the mRNA binding capacity by RNA Immunoprecipitation (RIP) (Figure S4A). In accordance with the literature demonstrating the direct binding of MYC mRNA by IGF2BP1 in various cellular settings [47]. We confirmed significantly enriched MYC RNA upon specific immobilization of IGF2BP1 protein (Figure 4B and Figure S4B). However, we also observed an accumulation of EZH2 mRNA in the IGF2BP1-immobilized fraction (Figure 4B and Figure S4B), supporting a direct interaction of EZH2 mRNA with IGF2BP1. As expected, there was no enrichment of negative control TERT mRNA compared to IgG control and immobilized IGF2BP1 (Figure 4B and Figure S4B). Additional mRNA decay experiments using the transcriptional repressor Actinomycin D revealed an increased destabilization of EZH2 mRNA upon depletion of IGF2BP1 (Figure 4C). These data suggest a potential role of IGF2BP1 in preventing miRNA-dependent decay of EZH2 mRNA. Recent reports described IGF2BP1 as a ‘reader’ of the N6-methyladenosine (m6A) modification of its target mRNAs, among them the serum response factor (SRF) [23]. Therefore, we analyzed the m6A-dependent regulation of EZH2 mRNA in NEN cells by depleting the adenosine-methyltransferases METTL3 and METTL14, which catalyze m6A. In fact, METTL3/14 knock-down resulted in decreased mRNA and protein levels of EZH2 in both BON1 and NCIH727 cells (Figure 4D and Figure S4C) suggesting a m6A-dependent interaction of EZH2 with IGF2BP1 which potentially inhibits EZH2 mRNA decay and thereby stabilizes protein levels. Moreover, the observed downregulation of SUZ12 upon IGF2BP1 knock-down is potentially also mediated by an m6A-dependent mechanism (Figure S4C,D). IGF2BP1 knock-down mediated reduction in EZH2 subsequently led to decreased levels of H3K27 trimethylation (Figure S4D). This is in line with other studies: Loss of EZH2 has been shown to induce cell cycle arrest in different solid cancer cells by reducing H3K27 trimethylation at promoters of cell cycle arrest genes such as p21, p27 and p16 [48,49,50]. Hence, upon IGF2BP1 knock-down we detected increased p27 levels in BON1 and NCIH272 cells (Figure S4D). Taken together, our data indicate that IGF2BP1 knock-down decreases EZH2 levels in NEN cells leads to numerous epigenetically induced upregulation of cell cycle checkpoints causing the observed phenotype. 

### 3.5. The IGF2BP1-MYC-EZH2 Network Is a Promising Therapeutic Target in NEN Cells

**Figure 5:**
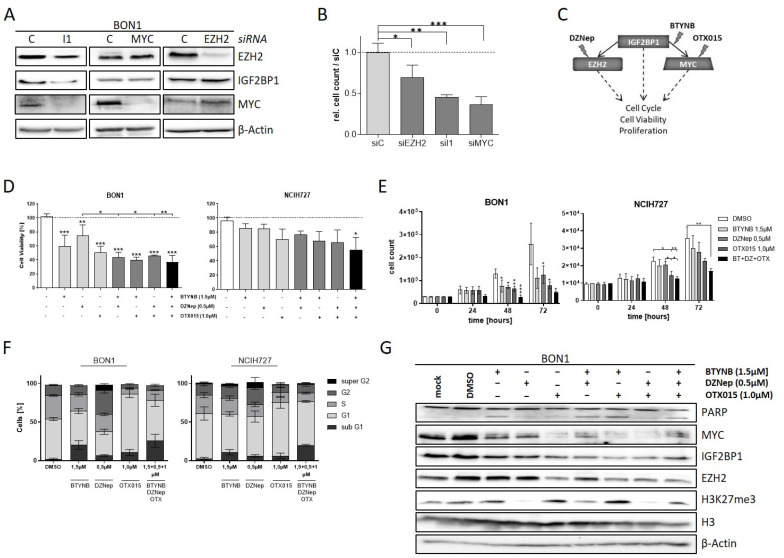
The IGF2BP1-MYC-EZH2 network is a target for pharmacological inhibition. (**A**) Representative Western blot of EZH2, IGF2BP1, and MYC knock-down in transfected BON1 cells. Actin served as loading control. (**B**) Proliferation of BON1 cells upon transfection with indicated siRNAs after 72 h. (**C**) Schematic strategy of IGF2BP1-MYC-EZH2 inhibition with commercially available inhibitors. DZNep targeting methylation activity of EZH2/PRC2 complex, BTYNB inhibiting IGF2BP1 binding to MYC mRNA and OTX015 inhibiting BRD2/3/4 transcription factors leading to reduced MYC expression. (**D**) Cell viability determined by Cell Titer Glo in BON1 and NCIH727 cells treated with indicated inhibitor concentrations for 72 h. Viability of DMSO treated cells served as control and was set to one. (**E**) Analysis of cell proliferation by cell counting at indicated time points upon treatment with indicated inhibitor concentrations. (**F**) Cell cycle analysis of NEN cells treated with indicated inhibitor concentrations for 72 h. Percentage of cells in indicated cell cycle phases were determined by propidium iodide staining. (**G**) Representative Western blot analysis of respective target proteins after treatment with indicated inhibitors for 72 h. Untreated (mock) and DMSO treated cells were used as expression controls. Actin served as loading control. Statistical significance was determined by Student’s *t*-test: * *p* < 0.05; ** *p* < 0.01; *** *p* < 0.001. The uncropped western blot figures were presented in Figure S7.

Downregulation of EZH2 and c-Myc potentially accounts for the phenotypic changes in NEN cells upon depletion of IGF2BP1. To validate this finding, we individually knocked-down the respective proteins in BON1 cells. As already described above, we observed decreased c-Myc and EZH2 expression upon knock-down of IGF2BP1 whereas knockdown of EZH2 had no influence on either c-Myc and IGF2BP1 protein levels (Figure 5A). Interestingly, although MYC is supposed to promote IGF2BP1 synthesis, depletion of c-Myc did not alter the protein expression of EZH2 and IGF2BP1 suggesting that IGF2BP1 downregulates EZH2 in NEN cells mainly independently of c-Myc. The knock-down of either protein led to decreased proliferation rates in BON1 cells highlighting the oncogenic impact of each protein (Figure 5B). Based on these data, we investigated different pharmacological strategies targeting distinct components of the IGF2BP1—c-Myc—EZH2 network individually or in combination (Figure 5C). For this purpose, we used BTYNB, a novel inhibitor of IGF2BP1 which interferes with the binding of Myc mRNA to IGF2BP1 protein, thus destabilizing Myc mRNA [51]. In addition, due to the lack of direct c-Myc inhibitors, we targeted c-Myc by using OTX015, a BET-inhibitor that reduces the transcription of MYC [52]. Targeting the PRC2 complex was carried out using DZNep, an inhibitor of histone-methyltransferases, which has already been described to inhibit EZH2 in numerous cell models in vitro [53,54]. Using these compounds individually or in combination, we analyzed the effects on cell viability and proliferation in the NEN cell lines (Figure 5D,E and Figure S5A). Consistent with our observation in knock-down experiments, all inhibitors had pronounced effects on viability and proliferation, with the BET-inhibitor OTX015 being the most potent agent (Figure 5D,E and Figure S5A). Moreover, combinatorial treatment synergistically decreased cell viability compared to single-agent treatment. The combination of all three inhibitors had the largest impact on cell viability and particularly proliferation (Figure 5D,E and Figure S5A). Further, analysis of cell cycle progression revealed different phenotypical changes associated with the applied inhibitors (Figure 5F and Figure S5A). Inhibition of IGF2BP1 binding to c-Myc RNA via BTYNB resulted in an increased sub-G1 population of cells (Figure 5F), which was confirmed by Annexin V staining (Figure S5B). This indicates a pro-apoptotic effect of the inhibitor, which, in fact, was different to our observations in IGF2BP1 knock-down experiments (Figure 2D) suggesting additional effects of BTYNB. Cells treated with the EZH2 inhibitor DZNep showed an increased G2-population and an additional ‘super G2′ peak suggesting a growth inhibitory effect caused by cellular stress potentially leading to polyploidy. The BET-inhibitor OTX015 led to a strong accumulation of cells in G1-phase of cell cycle. In contrast, simultaneous treatment with all three inhibitors resulted in a further increased sub-G1 phase indicating pronounced apoptotic cell death (Figure 5F). Analysis of protein expression confirmed the expected inhibitory mechanisms of action of the respective inhibitors. Hence, BTYNB and OTX015 decreased the expression of c-Myc, whereas DZNep led to reduced trimethylation of Lysine 27 on Histone 3 (Figure 5G). Additionally, c-Myc inhibition via OTX015 resulted in decreased levels of IGF2BP1 and subsequent reduced EZH2 expression, possibly due to a partial c-Myc-dependent feedback loop regulating the expression of IGF2BP1 [55]. PARP cleavage indicating apoptotic cell death was induced upon treatment with BTYNB (single or combinatorial) consistent with the phenotypical changes observed in both cell cycle and Annexin V analyses.

Taken together, our results suggest that targeting the IGF2BP1-c-Myc-EZH2 network at various levels might be a promising therapeutic approach in neuroendocrine tumors, potentially also by using combinatorial approaches, warranting further in vivo validation.

## 4. Discussion

In this report, we identified the mRNA-binding protein IGF2BP1 as an important post-transcriptional regulator of EZH2-driven cell proliferation in neuroendocrine tumors. Our findings are in line with recent reports that IGF2BP1 is able to stimulate tumor cell proliferation in vitro and in vivo by enhancing E2F-driven gene expression, thereby promoting G1/S transition [14]. E2F, in turn, is able to enhance EZH2 transcription in different human cancers by directly binding to the EZH2 promoter which facilitates cell cycle entry and S phase progression [56,57,58]. Consistently with these literature reports, we found that IGF2BP1 affects expression of EZH2 and its downstream effectors regulating cell proliferation, such as CDK2 and CCNE1 in neuroendocrine tumor cells, thereby driving G1/S transition.

IGF2BP1 has been described as a reader of N6-methyladenosine (m6A), a well-known post-transcriptional modification and the most common type of methylation in eukaryotic mRNAs [59]. It has been demonstrated before that IGF2BP1 enhances tumor cell proliferation through a m6A-dependent stabilization of MYC mRNA, one of its major known oncogenic targets [19,27,60]. Here, we show first evidence that IGF2BP1 directly also impairs the degradation of EZH2 mRNA in a m6A-dependent manner, resulting in elevated EZH2 transcriptional activity and enhanced cell cycle progression. This direct m6A-dependent stabilization of EZH2 mRNA by IGF2BP1 further underlines its oncogenic role in cancer, which so far has mainly been attributed to its inhibitory action on miRNA/RISC-directed downregulation of antiproliferative target mRNAs [14,17,30,61,62]. The hypothesis that stabilization of EZH2 mRNA by IGF2BP1 depends on METTL3/14 dependent m6A-methylation was corroborated by the fact that METTL3/14 depletion led to the reduced expression of EZH2, as described previously for the IGF2BP1-dependent stabilization of MYC mRNA [27]. This unravels IGF2BP1 as a post-transcriptional enhancer of EZH2-dependent cell proliferation, in addition to its already known transcriptional upregulation by the transcription factor E2F [14].

Previous studies showed that EZH2 expression is post-transcriptionally downregulated by various miRNAs that are able to bind to the 3′UTR of EZH2 mRNA. Subsequently, EZH2 protein levels, as well as consecutive accumulation of H3K27me3, are reduced, thereby impairing tumor progression, as demonstrated in colon, breast, hepatocellular, and nasopharyngeal cancers, as well as melanoma and glioblastoma [63]. These data imply that interfering with miRNAs targeting the EZH2 mRNA represents an interesting therapeutic option. Similarly to these findings, our data identify IGF2BP1 as a novel therapeutic target whose knockdown destabilizes EZH2 mRNA leading to reduced cell cycle progression in vitro.

EZH2 is highly expressed in various human cancers [56,64,65] including neuroendocrine tumors [66,67]. As well-described epigenetic regulator of cell cycle progression [37] and cell lineage determination [68], EZH2 overexpression correlates with aggressiveness and poor survival in prostate, colorectal, and breast cancers, as well as melanomas [69,70,71,72]. Furthermore, therapeutic EZH2 inhibition by either 3-deazaadenosine A (DZNep) or genetic knockdown has been shown to induce autophagy and apoptosis in colorectal cancer cells in vitro [70]. We confirmed that DZNep suppresses H3K27me3 leading to a reduced cell proliferation rate and cell viability in NEN cells. However, no induction of apoptosis could be detected upon DZNep treatment. These cell type-specific differences might be explained by the fact that DZNep affects global, not EZH2-specific histone methylation by inhibiting the S-adenosyl-homocysteine hydrolase, which leads to the accumulation of the inhibitory S-adenosyl-homocysteine with broad effects on different cellular pathways [69,73]. In line with our findings, a recent report using a different EZH2 inhibitor, GSK126, showed significant antitumoral effects induced by EZH2 inhibition both in vitro and in a murine model of pancreatic neuroendocrine tumorigenesis [74].

To target the IGF2BP1 signaling pathway directly, we utilized the recently published small molecule inhibitor BTYNB which interferes with IGF2BP1 binding to its target mRNAs, such as MYC, thereby impairing tumor cell proliferation [51]. In addition, BTYNB has been shown to affect IGF2BP1-dependent mRNA stabilization leading to reduced cancer progression in vitro and in vivo [14]. Our data demonstrate that BTYNB impairs cell proliferation and induces apoptosis in NEN cells, accompanied by reduced levels of the MYC target protein. Interestingly, expression of the target EZH2, as well as H3K27me3 remained unchanged upon BTYNB treatment, indicating that BTYNB primarily affects the EZH2-independent downstream pathways of IGF2BP1. In line with this notion, we could show that BTYNB acts in a synergistic manner with the EZH2-inhibitor DZNep in inducing apoptosis.

Therapeutic synergism between IGF2BP1 and EZH2 inhibition was further enhanced by direct inhibition of the downstream target MYC using the small molecule inhibitor OTX015. This combinatorial targeting led to a strict G1 arrest and increased apoptosis in NEN cells.

## 5. Conclusions

Taken together, our data provide novel preclinical evidence that combined pharmacological targeting of the IGF2BP1-EZH2-MYC axis at various levels offers a promising, yet unexplored, strategy for the therapy of neuroendocrine tumors which warrants further validation in clinical studies.

## Data Availability

All data generated or analyzed during this study are included in this published article and its supplementary information files.

## Supplementary Materials

The following supporting information can be downloaded at: https://www.mdpi.com/article/10.3390/cancers14092121/s1, Figure S1: IGF2BP1 expression in NEN cell lines; Figure S2: IGF2BP1 knockdown and apoptosis in NEN cell lines. Figure S3: IGF2BP1 knockdown affects PRC2 complex and network; Figure S4: IGF2BP1 regulates stability of PRC2 components; Figure S5: Pharmacological inhibition of IGF2BP1-MYC-EZH2 network; Figure S6: Pharmacological inhibition of IGF2BP1-MYC-EZH2 network on apoptosis. Table S1: siRNA; Table S2: RNA-seq data; Table S3: KEGG-pathway analysis; Table S4: qRT-PCR primer; Table S5: Antibodies. Figure S7. Uncropped western blot figures.

## References

[1] Dasari A., Shen C., Halperin D., Zhao B., Zhou S., Xu Y., Shih T., Yao J.C. (2017). Trends in the Incidence, Prevalence, and Survival Outcomes in Patients With Neuroendocrine Tumors in the United States. JAMA Oncol..

[2] Oronsky B., Ma P.C., Morgensztern D., Carter C.A. (2017). Nothing But NET: A Review of Neuroendocrine Tumors and Carcinomas. Neoplasia.

[3] Bosman F.T., Carneiro F., Hruban R.H., Theise N.D. (2010). WHO Classification of Tumours of the Digestive System.

[4] Asamura H., Kameya T., Matsuno Y., Noguchi M., Tada H., Ishikawa Y., Yokose T., Jiang S.X., Inoue T., Nakagawa K. (2006). Neuroendocrine neoplasms of the lung: A prognostic spectrum. J. Clin. Oncol..

[5] Lewis M.A., Hobday T.J. (2012). Treatment of neuroendocrine tumor liver metastases. Int. J. Hepatol..

[6] Alexandraki K.I., Karapanagioti A., Karoumpalis I., Boutzios G., Kaltsas G.A. (2017). Advances and Current Concepts in the Medical Management of Gastroenteropancreatic Neuroendocrine Neoplasms. BioMed Res. Int..

[7] Funakoshi S., Hashiguchi A., Teramoto K., Miyata N., Kurita S., Adachi M., Hamamoto Y., Higuchi H., Takaishi H., Hibi T. (2013). Second-line chemotherapy for refractory small cell neuroendocrine carcinoma of the esophagus that relapsed after complete remission with irinotecan plus cisplatin therapy: Case report and review of the literature. Oncol. Lett..

[8] Mafficini A., Scarpa A. (2018). Genomic landscape of pancreatic neuroendocrine tumours: The International Cancer Genome Consortium. J. Endocrinol..

[9] Jiao Y., Shi C., Edil B.H., de Wilde R.F., Klimstra D.S., Maitra A., Schulick R.D., Tang L.H., Wolfgang C.L., Choti M.A. (2011). DAXX/ATRX, MEN1, and mTOR pathway genes are frequently altered in pancreatic neuroendocrine tumors. Science.

[10] Scarpa A., Chang D.K., Nones K., Corbo V., Patch A.-M., Bailey P., Lawlor R.T., Johns A.L., Miller D.K., Mafficini A. (2017). Whole-genome landscape of pancreatic neuroendocrine tumours. Nature.

[11] Di Domenico A., Wiedmer T., Marinoni I., Perren A. (2017). Genetic and epigenetic drivers of neuroendocrine tumours (NET). Endocr.-Relat. Cancer.

[12] Lin W., Watanabe H., Peng S., Francis J.M., Kaplan N., Pedamallu C.S., Ramachandran A., Agoston A., Bass A.J., Meyerson M. (2015). Dynamic epigenetic regulation by menin during pancreatic islet tumor formation. Mol. Cancer Res..

[13] Mafficini A., Scarpa A. (2019). Genetics and Epigenetics of Gastroenteropancreatic Neuroendocrine Neoplasms. Endocr. Rev..

[14] Muller S., Bley N., Busch B., Glass M., Lederer M., Misiak C., Fuchs T., Wedler A., Haase J., Bertoldo J.B. (2020). The oncofetal RNA-binding protein IGF2BP1 is a druggable, post-transcriptional super-enhancer of E2F-driven gene expression in cancer. Nucleic Acids Res..

[15] Bell J.L., Wächter K., Mühleck B., Pazaitis N., Köhn M., Lederer M., Hüttelmaier S. (2013). Insulin-like growth factor 2 mRNA-binding proteins (IGF2BPs): Post-transcriptional drivers of cancer progression?. Cell. Mol. Life Sci. CMLS.

[16] Lederer M., Bley N., Schleifer C., Hüttelmaier S. (2014). The role of the oncofetal IGF2 mRNA-binding protein 3 (IGF2BP3) in cancer. Semin. Cancer Biol..

[17] Muller S., Bley N., Glass M., Busch B., Rousseau V., Misiak D., Fuchs T., Lederer M., Huttelmaier S. (2018). IGF2BP1 enhances an aggressive tumor cell phenotype by impairing miRNA-directed downregulation of oncogenic factors. Nucleic Acids Res..

[18] Bell J.L., Turlapati R., Liu T., Schulte J.H., Hüttelmaier S. (2015). IGF2BP1 harbors prognostic significance by gene gain and diverse expression in neuroblastoma. J. Clin. Oncol. Off. J. Am. Soc. Clin. Oncol..

[19] Gutschner T., Hammerle M., Pazaitis N., Bley N., Fiskin E., Uckelmann H., Heim A., Grobeta M., Hofmann N., Geffers R. (2014). Insulin-like growth factor 2 mRNA-binding protein 1 (IGF2BP1) is an important protumorigenic factor in hepatocellular carcinoma. Hepatology.

[20] Ghoshal A., Rodrigues L.C., Gowda C.P., Elcheva I.A., Liu Z., Abraham T., Spiegelman V.S. (2019). Extracellular vesicle-dependent effect of RNA-binding protein IGF2BP1 on melanoma metastasis. Oncogene.

[21] Zirkel A., Lederer M., Stohr N., Pazaitis N., Huttelmaier S. (2013). IGF2BP1 promotes mesenchymal cell properties and migration of tumor-derived cells by enhancing the expression of LEF1 and SNAI2 (SLUG). Nucleic Acids Res..

[22] Elcheva I.A., Wood T., Chiarolanzio K., Chim B., Wong M., Singh V., Gowda C.P., Lu Q., Hafner M., Dovat S. (2020). RNA-binding protein IGF2BP1 maintains leukemia stem cell properties by regulating HOXB4, MYB, and ALDH1A1. Leukemia.

[23] Muller S., Glass M., Singh A.K., Haase J., Bley N., Fuchs T., Lederer M., Dahl A., Huang H., Chen J. (2019). IGF2BP1 promotes SRF-dependent transcription in cancer in a m6A- and miRNA-dependent manner. Nucleic Acids Res..

[24] Faye M.D., Beug S.T., Graber T.E., Earl N., Xiang X., Wild B., Langlois S., Michaud J., Cowan K.N., Korneluk R.G. (2015). IGF2BP1 controls cell death and drug resistance in rhabdomyosarcomas by regulating translation of cIAP1. Oncogene.

[25] Weidensdorfer D., Stöhr N., Baude A., Lederer M., Köhn M., Schierhorn A., Buchmeier S., Wahle E., Hüttelmaier S. (2009). Control of c-myc mRNA stability by IGF2BP1-associated cytoplasmic RNPs. RNA.

[26] Rickman D.S., Schulte J.H., Eilers M. (2018). The Expanding World of N-MYC-Driven Tumors. Cancer Discov..

[27] Huang H., Weng H., Sun W., Qin X., Shi H., Wu H., Zhao B.S., Mesquita A., Liu C., Yuan C.L. (2018). Recognition of RNA N(6)-methyladenosine by IGF2BP proteins enhances mRNA stability and translation. Nat. Cell Biol..

[28] Berthon C., Raffoux E., Thomas X., Vey N., Gomez-Roca C., Yee K., Taussig D.C., Rezai K., Roumier C., Herait P. (2016). Bromodomain inhibitor OTX015 in patients with acute leukaemia: A dose-escalation, phase 1 study. Lancet Haematol..

[29] Doroshow D.B., Eder J.P., LoRusso P.M. (2017). BET inhibitors: A novel epigenetic approach. Ann. Oncol..

[30] Sparanese D., Lee C.H. (2007). CRD-BP shields c-myc and MDR-1 RNA from endonucleolytic attack by a mammalian endoribonuclease. Nucleic Acids Res..

[31] Busch B., Bley N., Muller S., Glass M., Misiak D., Lederer M., Vetter M., Strauss H.G., Thomssen C., Huttelmaier S. (2016). The oncogenic triangle of HMGA2, LIN28B and IGF2BP1 antagonizes tumor-suppressive actions of the let-7 family. Nucleic Acids Res..

[32] Keller M.P., Choi Y., Wang P., Davis D.B., Rabaglia M.E., Oler A.T., Stapleton D.S., Argmann C., Schueler K.L., Edwards S. (2008). A gene expression network model of type 2 diabetes links cell cycle regulation in islets with diabetes susceptibility. Genome Res..

[33] Drexler H.G., Uphoff C.C. (2002). Mycoplasma contamination of cell cultures: Incidence, sources, effects, detection, elimination, prevention. Cytotechnology.

[34] Hanahan D. (1985). Heritable formation of pancreatic ß-cell tumours in transgenic mice expressing recombinant insulin/simian virus 40 oncogenes. Nature.

[35] Scott A.T., Weitz M., Breheny P.J., Ear P.H., Darbro B., Brown B.J., Braun T.A., Li G., Umesalma S., Kaemmer C.A. (2020). Gene Expression Signatures Identify Novel Therapeutics for Metastatic Pancreatic Neuroendocrine Tumors. Clin. Cancer Res..

[36] Garcia-Gutierrez L., Bretones G., Molina E., Arechaga I., Symonds C., Acosta J.C., Blanco R., Fernandez A., Alonso L., Sicinski P. (2019). Myc stimulates cell cycle progression through the activation of Cdk1 and phosphorylation of p27. Sci Rep..

[37] Comet I., Riising E.M., Leblanc B., Helin K. (2016). Maintaining cell identity: PRC2-mediated regulation of transcription and cancer. Nat. Rev. Cancer.

[38] Koh C.M., Iwata T., Zheng Q., Bethel C., Yegnasubramanian S., De Marzo A.M. (2011). Myc enforces overexpression of EZH2 in early prostatic neoplasia via transcriptional and post-transcriptional mechanisms. Oncotarget.

[39] Yang R., Wang M., Zhang G., Bao Y., Wu Y., Li X., Yang W., Cui H. (2020). E2F7-EZH2 axis regulates PTEN/AKT/mTOR signalling and glioblastoma progression. Br. J. Cancer.

[40] Yamaguchi H., Hung M.C. (2014). Regulation and Role of EZH2 in Cancer. Cancer Res. Treat..

[41] Lu W., Liu S., Li B., Xie Y., Izban M.G., Ballard B.R., Sathyanarayana S.A., Adunyah S.E., Matusik R.J., Chen Z. (2017). SKP2 loss destabilizes EZH2 by promoting TRAF6-mediated ubiquitination to suppress prostate cancer. Oncogene.

[42] Chu C.S., Lo P.W., Yeh Y.H., Hsu P.H., Peng S.H., Teng Y.C., Kang M.L., Wong C.H., Juan L.J. (2014). O-GlcNAcylation regulates EZH2 protein stability and function. Proc. Natl. Acad. Sci. USA.

[43] Xu J.H., Hu S.L., Shen G.D., Shen G. (2016). Tumor suppressor genes and their underlying interactions in paclitaxel resistance in cancer therapy. Cancer Cell Int..

[44] Benetatos L., Vartholomatos G., Hatzimichael E. (2014). Polycomb group proteins and MYC: The cancer connection. Cell. Mol. Life Sci..

[45] Salvatori B., Iosue I., Djodji Damas N., Mangiavacchi A., Chiaretti S., Messina M., Padula F., Guarini A., Bozzoni I., Fazi F. (2011). Critical Role of c-Myc in Acute Myeloid Leukemia Involving Direct Regulation of miR-26a and Histone Methyltransferase EZH2. Genes Cancer.

[46] Neri F., Zippo A., Krepelova A., Cherubini A., Rocchigiani M., Oliviero S. (2012). Myc regulates the transcription of the PRC2 gene to control the expression of developmental genes in embryonic stem cells. Mol. Cell. Biol..

[47] Lemm I., Ross J. (2002). Regulation of c-myc mRNA decay by translational pausing in a coding region instability determinant. Mol. Cell. Biol..

[48] Fan T., Jiang S., Chung N., Alikhan A., Ni C., Lee C.C., Hornyak T.J. (2011). EZH2-dependent suppression of a cellular senescence phenotype in melanoma cells by inhibition of p21/CDKN1A expression. Mol. Cancer Res..

[49] Ougolkov A.V., Bilim V.N., Billadeau D.D. (2008). Regulation of pancreatic tumor cell proliferation and chemoresistance by the histone methyltransferase enhancer of zeste homologue 2. Clin. Cancer Res..

[50] Ito T., Teo Y.V., Evans S.A., Neretti N., Sedivy J.M. (2018). Regulation of Cellular Senescence by Polycomb Chromatin Modifiers through Distinct DNA Damage- and Histone Methylation-Dependent Pathways. Cell Rep..

[51] Mahapatra L., Andruska N., Mao C., Le J., Shapiro D.J. (2017). A Novel IMP1 Inhibitor, BTYNB, Targets c-Myc and Inhibits Melanoma and Ovarian Cancer Cell Proliferation. Transl. Oncol..

[52] Coude M.M., Braun T., Berrou J., Dupont M., Bertrand S., Masse A., Raffoux E., Itzykson R., Delord M., Riveiro M.E. (2015). BET inhibitor OTX015 targets BRD2 and BRD4 and decreases c-MYC in acute leukemia cells. Oncotarget.

[53] Puppe J., Drost R., Liu X., Joosse S.A., Evers B., Cornelissen-Steijger P., Nederlof P., Yu Q., Jonkers J., van Lohuizen M. (2009). BRCA1-deficient mammary tumor cells are dependent on EZH2 expression and sensitive to Polycomb Repressive Complex 2-inhibitor 3-deazaneplanocin A. Breast Cancer Res..

[54] Tan J., Yang X., Zhuang L., Jiang X., Chen W., Lee P.L., Karuturi R.K., Tan P.B., Liu E.T., Yu Q. (2007). Pharmacologic disruption of Polycomb-repressive complex 2-mediated gene repression selectively induces apoptosis in cancer cells. Genes Dev..

[55] Noubissi F.K., Nikiforov M.A., Colburn N., Spiegelman V.S. (2010). Transcriptional Regulation of CRD-BP by c-myc: Implications for c-myc Functions. Genes Cancer.

[56] Bracken A.P., Pasini D., Capra M., Prosperini E., Colli E., Helin K. (2003). EZH2 is downstream of the pRB-E2F pathway, essential for proliferation and amplified in cancer. EMBO J..

[57] Kalashnikova E.V., Revenko A.S., Gemo A.T., Andrews N.P., Tepper C.G., Zou J.X., Cardiff R.D., Borowsky A.D., Chen H.W. (2010). ANCCA/ATAD2 overexpression identifies breast cancer patients with poor prognosis, acting to drive proliferation and survival of triple-negative cells through control of B-Myb and EZH2. Cancer Res..

[58] Duan Z., Zou J.X., Yang P., Wang Y., Borowsky A.D., Gao A.C., Chen H.W. (2013). Developmental and androgenic regulation of chromatin regulators EZH2 and ANCCA/ATAD2 in the prostate Via MLL histone methylase complex. Prostate.

[59] Zhao Y., Shi Y., Shen H., Xie W. (2020). m(6)A-binding proteins: The emerging crucial performers in epigenetics. J. Hematol. Oncol..

[60] Köbel M., Weidensdorfer D., Reinke C., Lederer M., Schmitt W.D., Zeng K., Thomssen C., Hauptmann S., Hüttelmaier S. (2007). Expression of the RNA-binding protein IMP1 correlates with poor prognosis in ovarian carcinoma. Oncogene.

[61] Elcheva I., Goswami S., Noubissi F.K., Spiegelman V.S. (2009). CRD-BP protects the coding region of betaTrCP1 mRNA from miR-183-mediated degradation. Mol. Cell.

[62] Jonson L., Christiansen J., Hansen T.V.O., Vikesa J., Yamamoto Y., Nielsen F.C. (2014). IMP3 RNP safe houses prevent miRNA-directed HMGA2 mRNA decay in cancer and development. Cell Rep..

[63] Gan L., Yang Y., Li Q., Feng Y., Liu T., Guo W. (2018). Epigenetic regulation of cancer progression by EZH2: From biological insights to therapeutic potential. Biomark. Res..

[64] Pasini D., Di Croce L. (2016). Emerging roles for Polycomb proteins in cancer. Curr. Opin. Genet. Dev..

[65] Yamagishi M., Uchimaru K. (2017). Targeting EZH2 in cancer therapy. Curr. Opin. Oncol..

[66] Faviana P., Marconcini R., Ricci S., Galli L., Lippolis P., Farci F., Castagna M., Boldrini L. (2019). EZH2 Expression in Intestinal Neuroendocrine Tumors. Appl. Immunohistochem. Mol. Morphol..

[67] Barazeghi E., Hellman P., Norlen O., Westin G., Stalberg P. (2021). EZH2 presents a therapeutic target for neuroendocrine tumors of the small intestine. Sci. Rep..

[68] Sparmann A., van Lohuizen M. (2006). Polycomb silencers control cell fate, development and cancer. Nat. Rev. Cancer.

[69] Varambally S., Dhanasekaran S.M., Zhou M., Barrette T.R., Kumar-Sinha C., Sanda M.G., Ghosh D., Pienta K.J., Sewalt R.G., Otte A.P. (2002). The polycomb group protein EZH2 is involved in progression of prostate cancer. Nature.

[70] Yao Y., Hu H., Yang Y., Zhou G., Shang Z., Yang X., Sun K., Zhan S., Yu Z., Li P. (2016). Downregulation of Enhancer of Zeste Homolog 2 (EZH2) is essential for the Induction of Autophagy and Apoptosis in Colorectal Cancer Cells. Genes.

[71] Zingg D., Debbache J., Schaefer S.M., Tuncer E., Frommel S.C., Cheng P., Arenas-Ramirez N., Haeusel J., Zhang Y., Bonalli M. (2015). The epigenetic modifier EZH2 controls melanoma growth and metastasis through silencing of distinct tumour suppressors. Nat. Commun..

[72] Mahara S., Lee P.L., Feng M., Tergaonkar V., Chng W.J., Yu Q. (2016). HIFI-alpha activation underlies a functional switch in the paradoxical role of Ezh2/PRC2 in breast cancer. Proc. Natl. Acad. Sci. USA.

[73] Zoabi M., Sadeh R., de Bie P., Marquez V.E., Ciechanover A. (2011). PRAJA1 is a ubiquitin ligase for the polycomb repressive complex 2 proteins. Biochem. Biophys. Res. Commun..

[74] April-Monn S.L., Andreasi V., Schiavo Lena M., Sadowski M.C., Kim-Fuchs C., Buri M.C., Ketkar A., Maire R., Di Domenico A., Schrader J. (2021). EZH2 Inhibition as New Epigenetic Treatment Option for Pancreatic Neuroendocrine Neoplasms (PanNENs). Cancers.

