# Supplementary material for: IGF2BP1 Promotes Proliferation of Neuroendocrine Neoplasms by Post-Transcriptional Enhancement of EZH2
Source: Cancers (Basel). 2022 Apr 24;14(9):2121. doi: 10.3390/cancers14092121 (PMC9131133; doi:10.3390/cancers14092121)
Supplement: Supplementary file 1 [file cancers-14-02121-s001.zip › cancers-1677009-Supplementary Materials/Supplementary Figures and Legends with S7.pdf]

Figure S1

A

Cell line	Origin
BON1	PNEN, Lymph Node metastasis
Colo320	Colon adenocarcinoma
NCIH727	Lung carcinoid tumor
QGP1	PNEN, primary

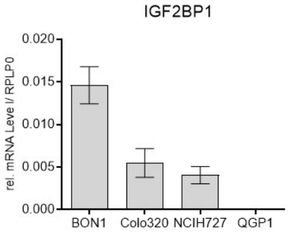

B

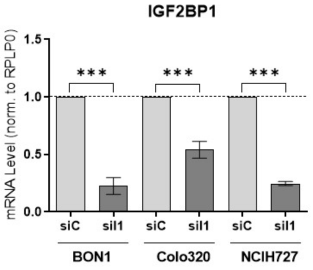

C

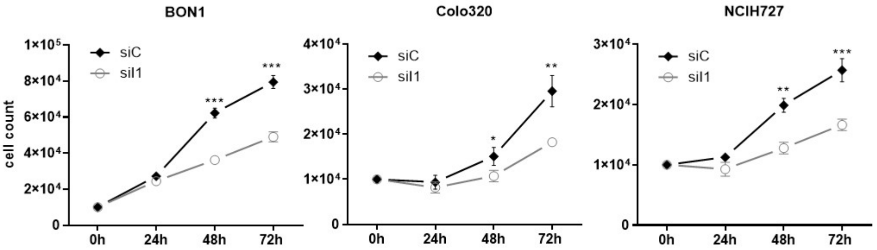

D

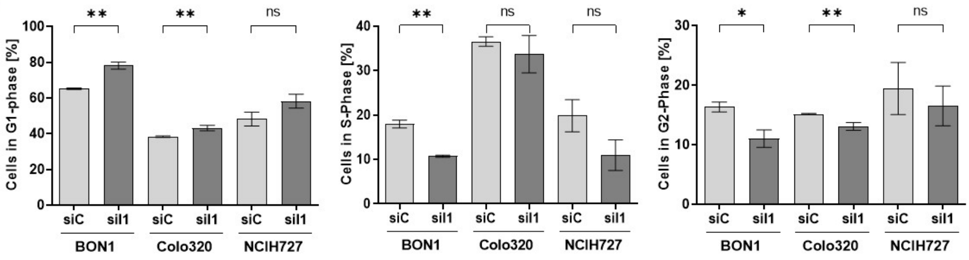

Figure S2

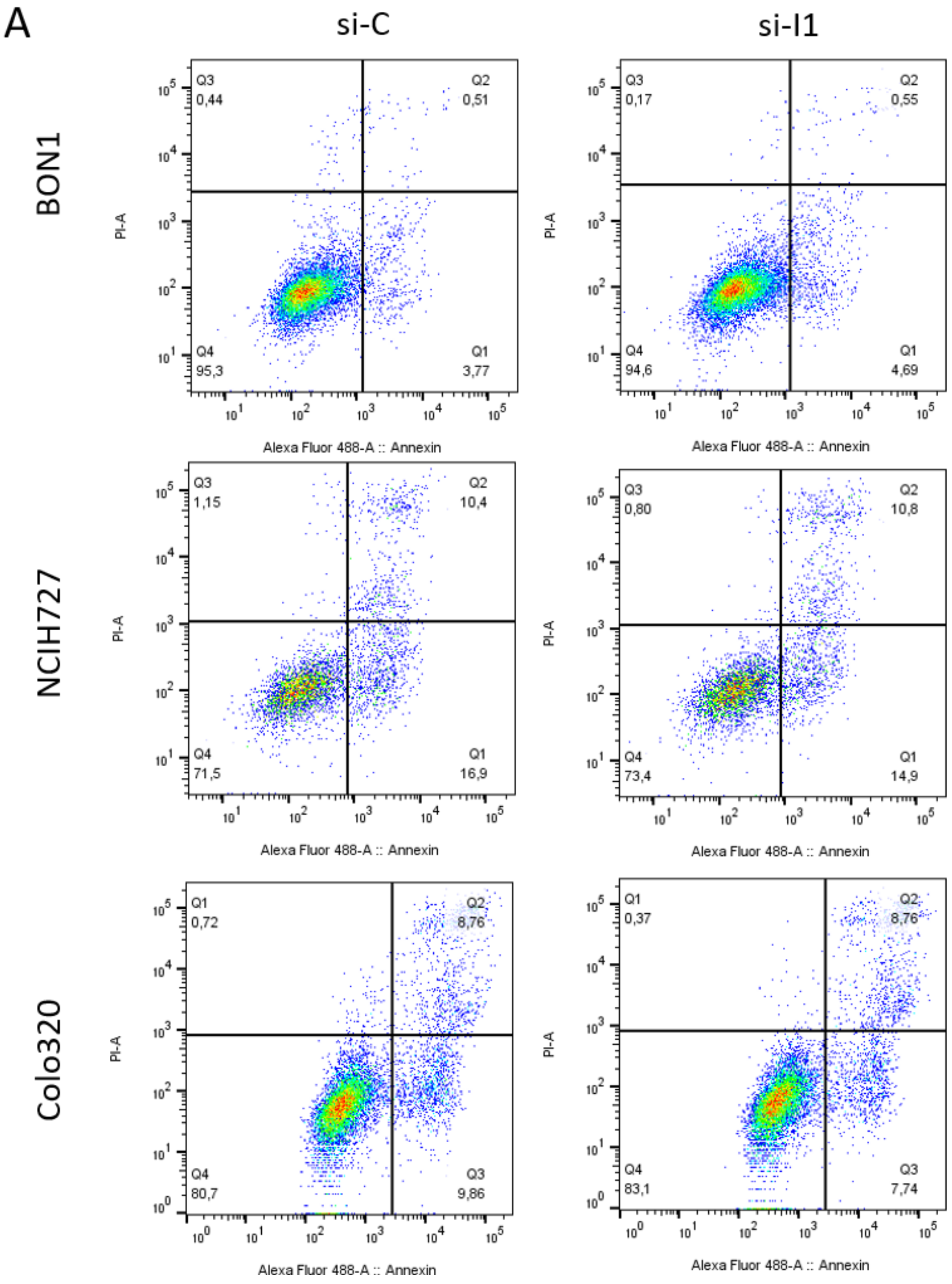
A
si-C
si-I1
BON1
NCIH727
Colo320
PI-A
Alexa Fluor 488-A :: Annexin
Q3 0,44
Q2 0,51
Q4 95,3
Q1 3,77
Q3 0,17
Q2 0,55
Q4 94,6
Q1 4,69
Q3 1,15
Q2 10,4
Q4 71,5
Q1 16,9
Q3 0,80
Q2 10,8
Q4 73,4
Q1 14,9
Q1 0,72
Q2 8,76
Q4 80,7
Q3 9,86
Q1 0,37
Q2 8,76
Q4 83,1
Q3 7,74

Figure S3

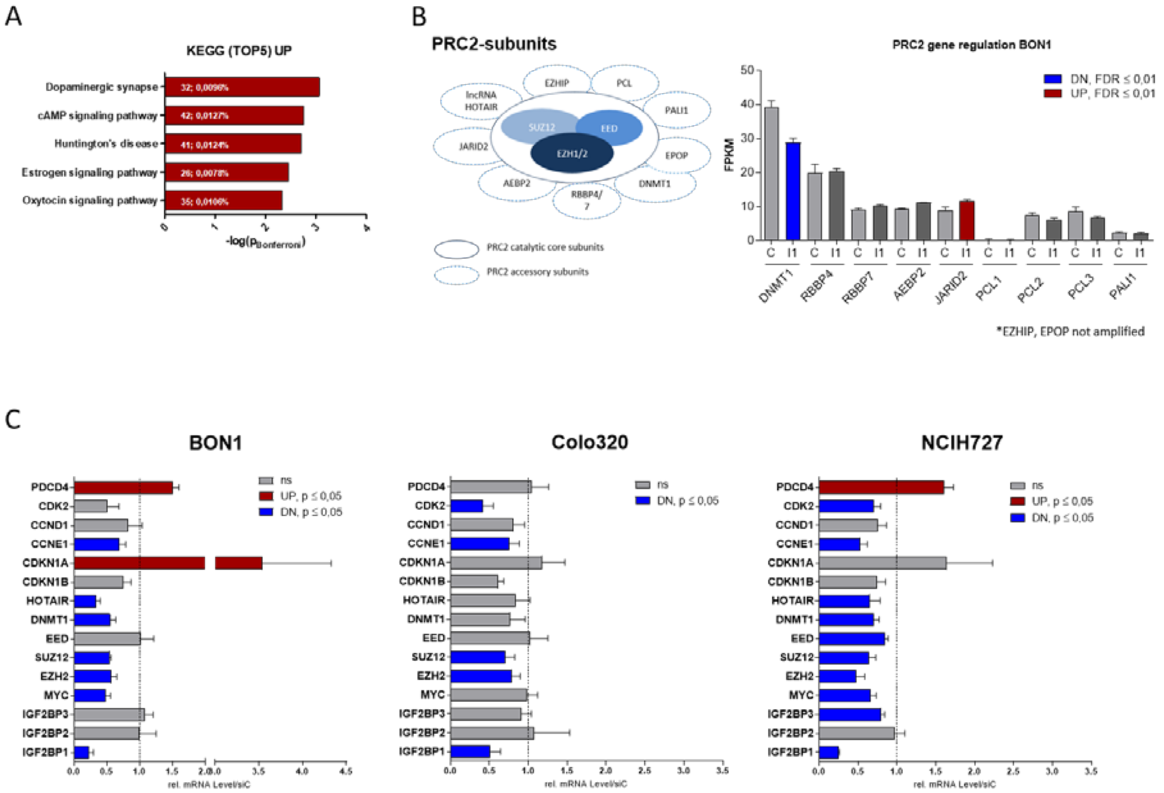
A
KEGG (TOP5) UP
Dopaminergic synapse
cAMP signaling pathway
Huntington's disease
Estrogen signaling pathway
Oxytocin signaling pathway
-log(pBonferroni)
B
PRC2-subunits
EZHIP
PCL
lncRNA HOTAIR
PALI1
SUZ12
EED
EZH1/2
JARID2
EPOP
AEBP2
RBBP4/7
DNMT1
PRC2 catalytic core subunits
PRC2 accessory subunits
PRC2 gene regulation BON1
DN, FDR ≤ 0,01
UP, FDR ≤ 0,01
FPKM
DNMT1
RBBP4
RBBP7
AEBP2
JARID2
PCL1
PCL2
PCL3
PALI1
*EZHIP, EPOP not amplified
C
BON1
Colo320
NCIH727
ns
UP, p ≤ 0,05
DN, p ≤ 0,05
PDCD4
CDK2
CCND1
CCNE1
CDKN1A
CDKN1B
HOTAIR
DNMT1
EED
SUZ12
EZH2
MYC
IGF2BP3
IGF2BP2
IGF2BP1
rel. mRNA Level/siC

Figure S4

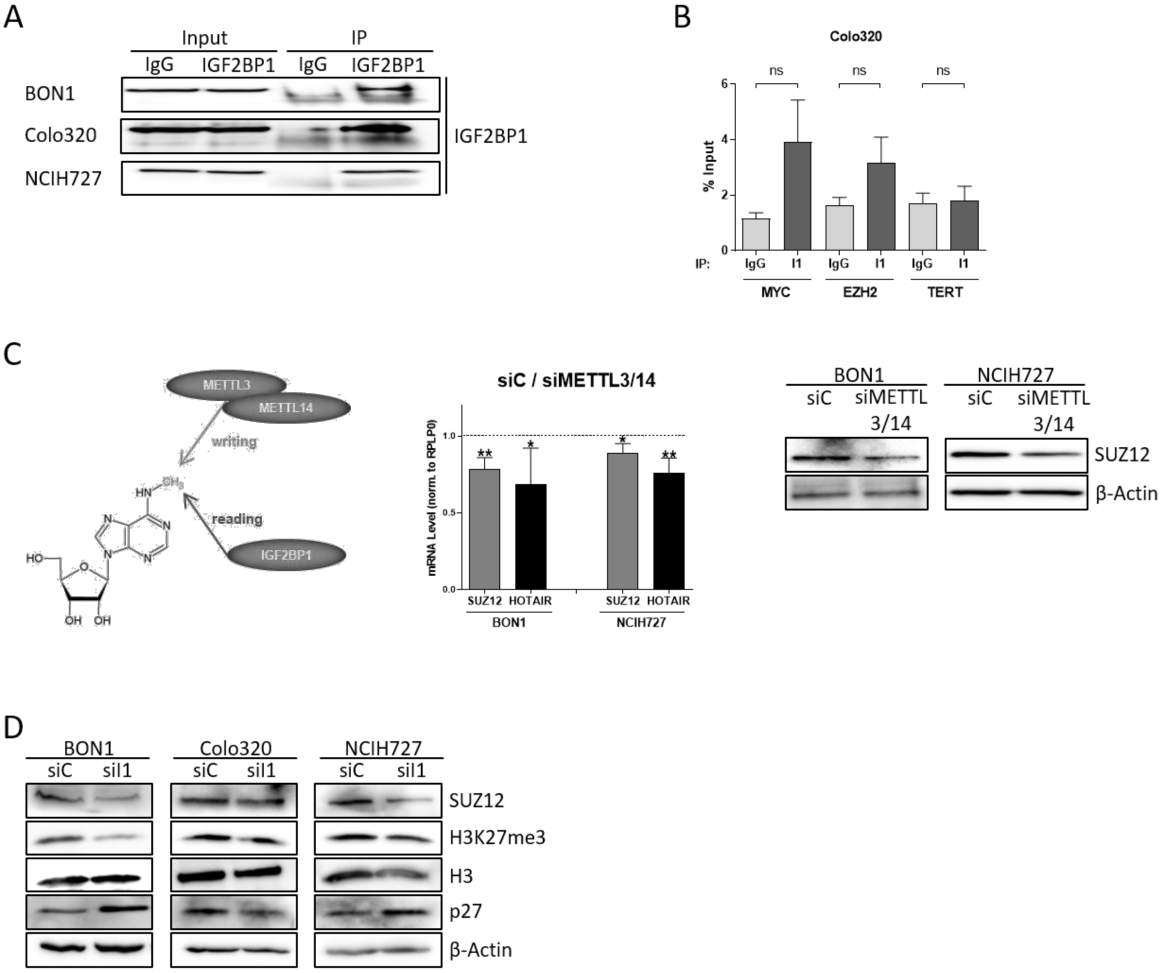

A
Input
IP
IgG
IGF2BP1
IgG
IGF2BP1
BON1
Colo320
NCIH727
IGF2BP1
B
Colo320
ns
ns
ns
% Input
6
4
2
0
IP:
IgG
I1
IgG
I1
IgG
I1
MYC
EZH2
TERT
C
METTL3
METTL14
writing
reading
IGF2BP1
HN
HO
OH
OH
siC / siMETTL3/14
mRNA Level (norm. to RPLP0)
1.0
0.5
0.0
**
*
*
**
SUZ12
HOTAIR
SUZ12
HOTAIR
BON1
NCIH727
BON1
NCIH727
siC
siMETTL 3/14
siC
siMETTL 3/14
SUZ12
β-Actin
D
BON1
Colo320
NCIH727
siC
siI1
siC
siI1
siC
siI1
SUZ12
H3K27me3
H3
p27
β-Actin

Figure S5

A

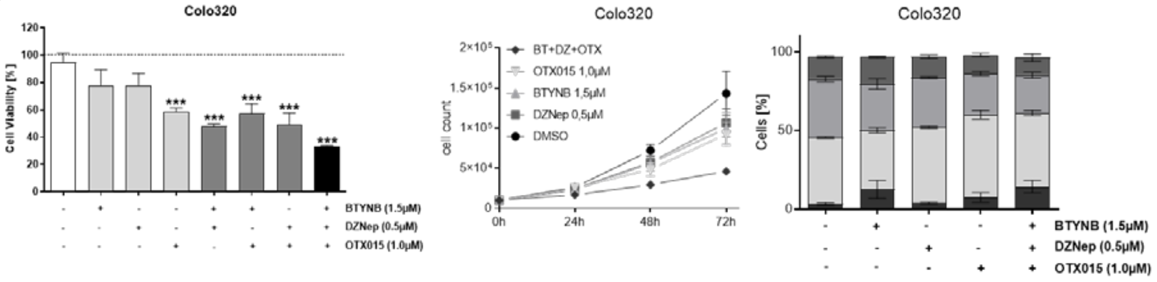
Colo320
Cell Viability [%]
120
100
80
60
40
20
0
***
BTYNB (1.5µM)
DZNep (0.5µM)
OTX015 (1.0µM)
Colo320
cell count
BT+DZ+OTX
OTX015 1,0µM
BTYNB 1,5µM
DZNep 0,5µM
DMSO
0h
24h
48h
72h
Colo320
Cells [%]
100
50
0
BTYNB (1.5µM)
DZNep (0.5µM)
OTX015 (1.0µM)

B

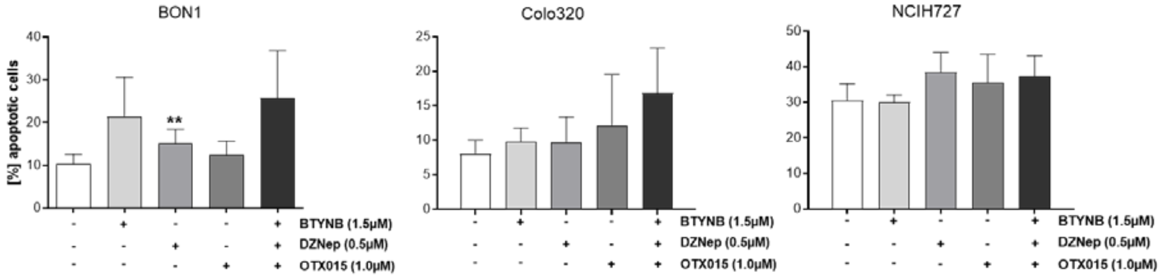
BON1
[%] apoptotic cells
**
BTYNB (1.5µM)
DZNep (0.5µM)
OTX015 (1.0µM)
Colo320
BTYNB (1.5µM)
DZNep (0.5µM)
OTX015 (1.0µM)
NCIH727
BTYNB (1.5µM)
DZNep (0.5µM)
OTX015 (1.0µM)

Figure S6

A

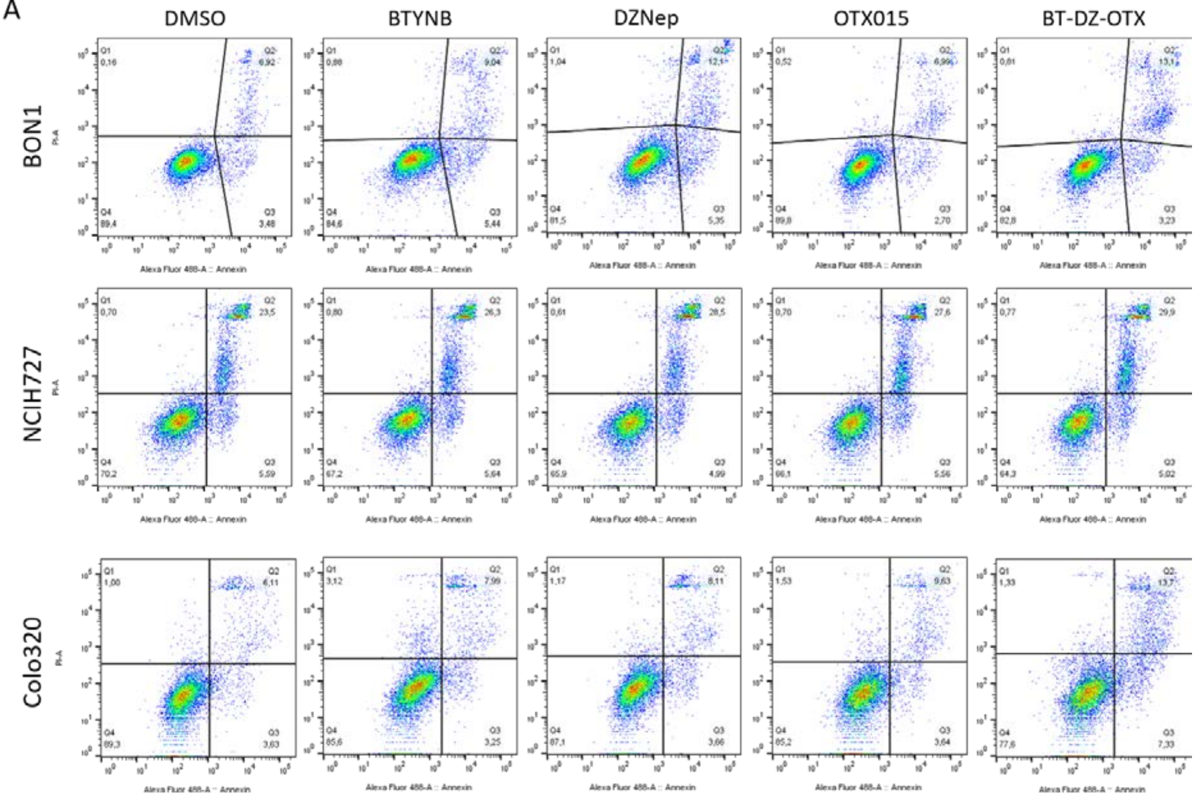

Figure S7: Uncropped Western Blot images used in this study.

Figure 1C. Western blot of IGF2BP1 protein levels in pooled benign murine pancreatic islet (BL6) and individual insulinoma of RIP1-Tag2 mice. Actin served as loading control. Leftmost lanes were loaded with PageRuler™ Prestained Protein Ladder. Molecular weights are in KDa units.

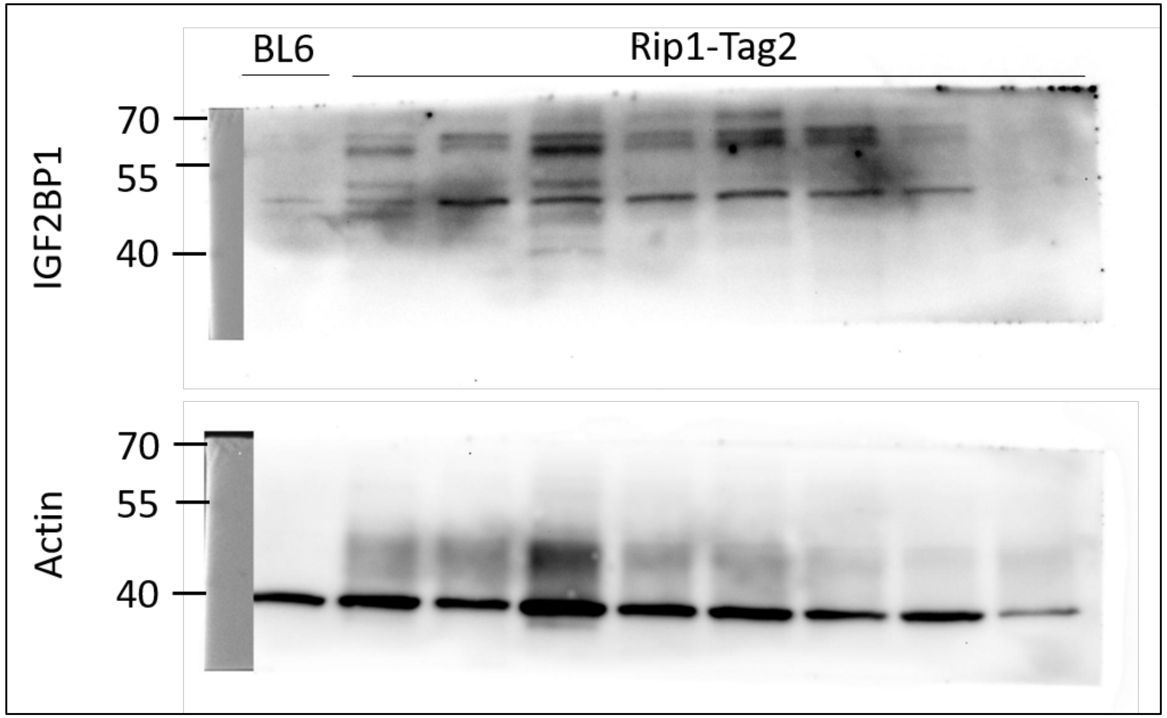

Figure 1D. Western blot of whole cell lysates from BON1, Colo320 and NCIH727 NEN cell lines. Actin served as loading control. Leftmost lanes were loaded with PageRuler™ Prestained Protein Ladder. Molecular weights are in KDa units.

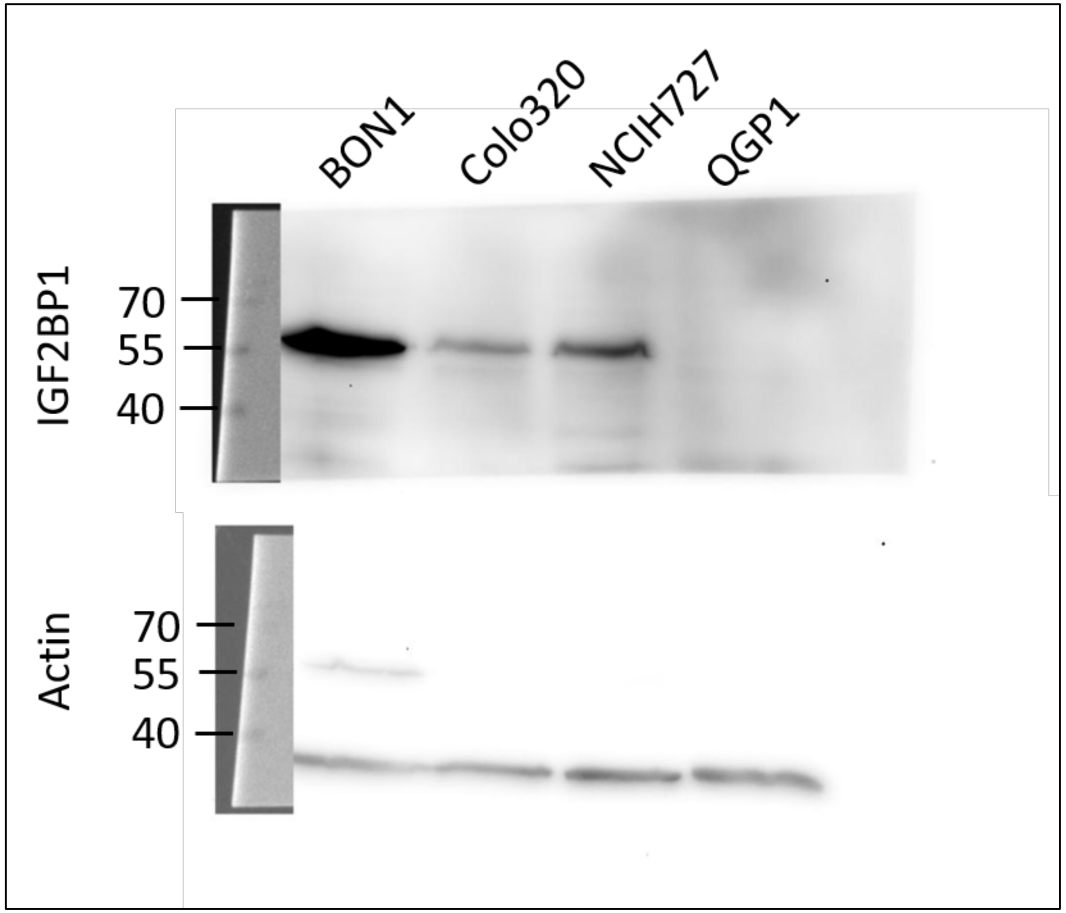

Figure 2A. Western blot of IGF2BP1 knockdown (siI1) in indicated NEN cell lines BON1, Colo320 and NCIH727 after 72 hours. Actin served as loading control. Leftmost and rightmost lanes were loaded with PageRuler™ Prestained Protein Ladder. Molecular weights are in KDa units.

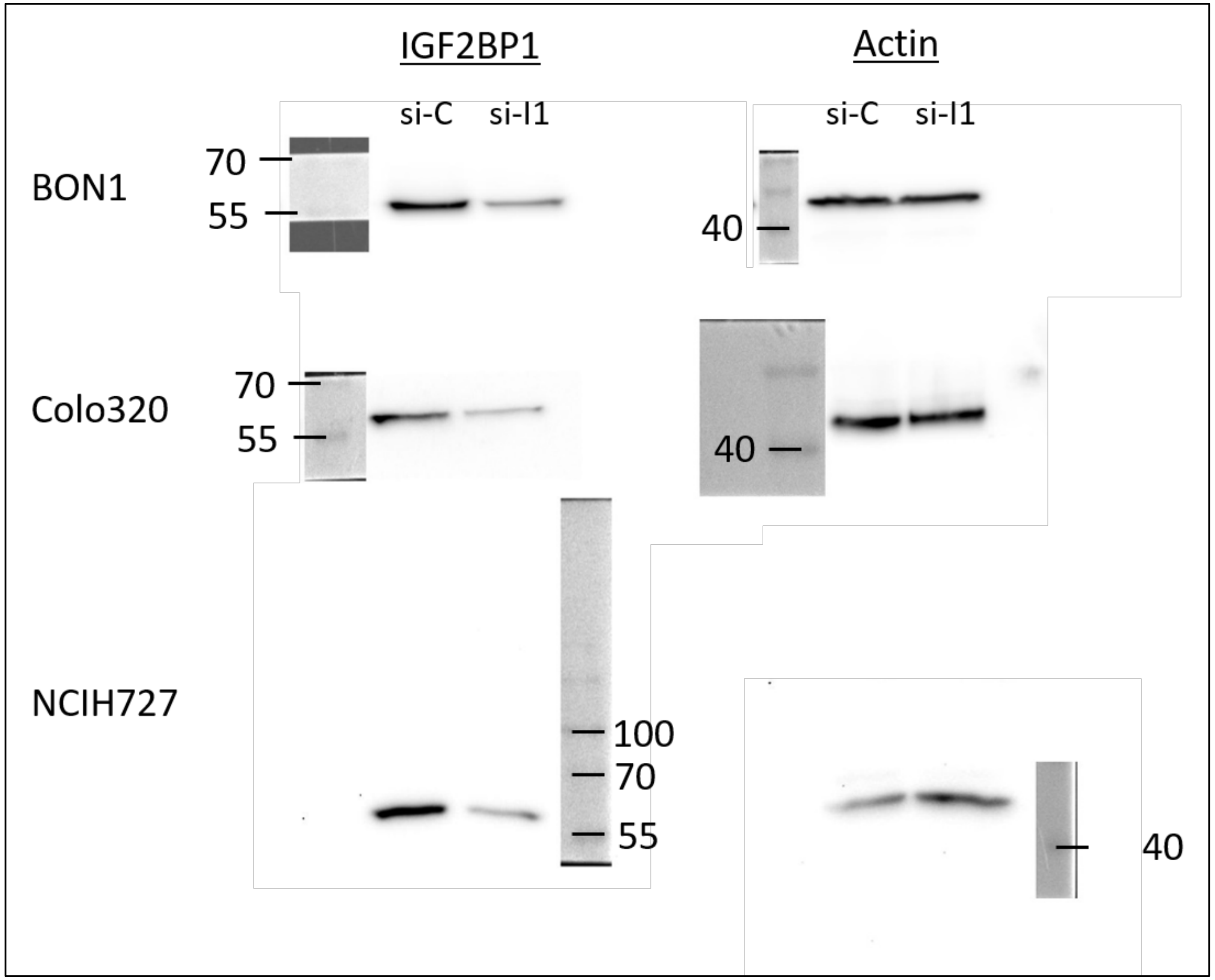

Figure 4A. Western blot of IGF2BP1, EZH2 and MYC upon knockdown of IGF2BP1 (siI1) in NEN cells BON1, Colo320 and NCIH727. Actin served as loading control. Leftmost and rightmost lanes were loaded with PageRuler™ Prestained Protein Ladder. Molecular weights are in KDa units.

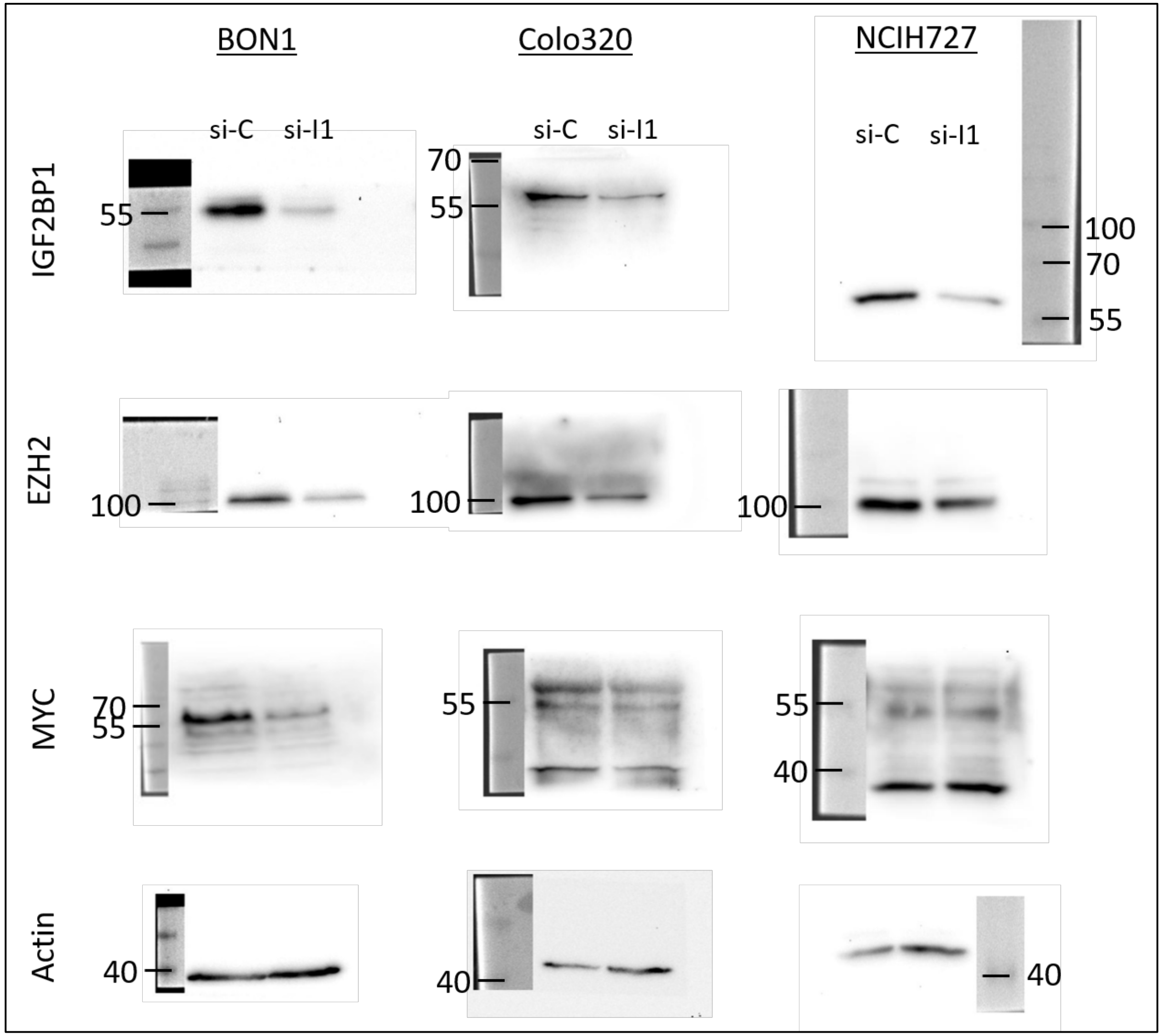

Figure 4D. Western blot of EZH2, METTL3 and METL14 after knockdown of METTL3/14 in BN1 and NCIH727 cells. Actin served as loading control. Leftmost lanes were loaded with PageRuler™ Prestained Protein Ladder. Molecular weights are in KDa units.

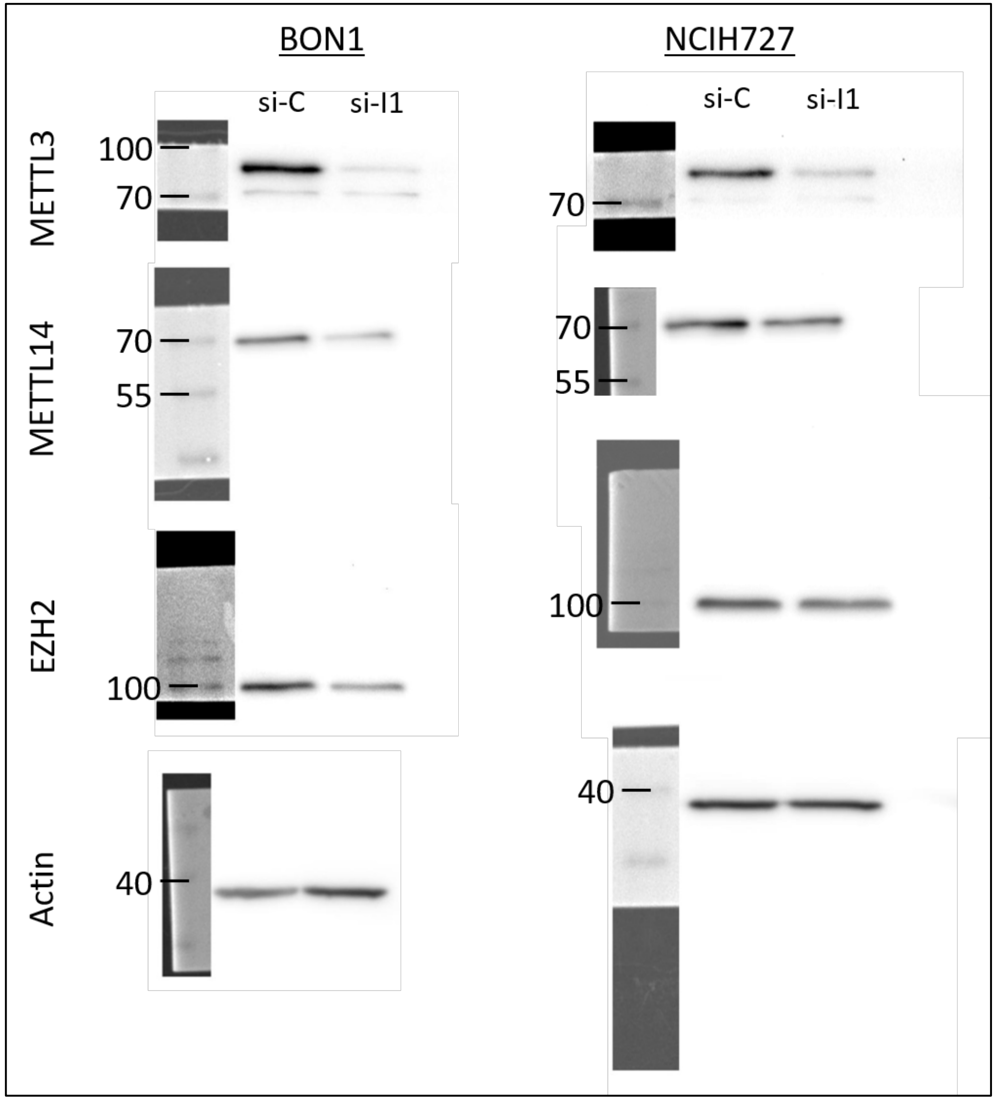

Figure 5A. Western blot of EZH2, IGF2BP1 and MYC after knockdown in transfected BON1 cells. . Actin served as loading control. Leftmost lanes were loaded with PageRuler™ Prestained Protein Ladder. Molecular weights are in KDa units.

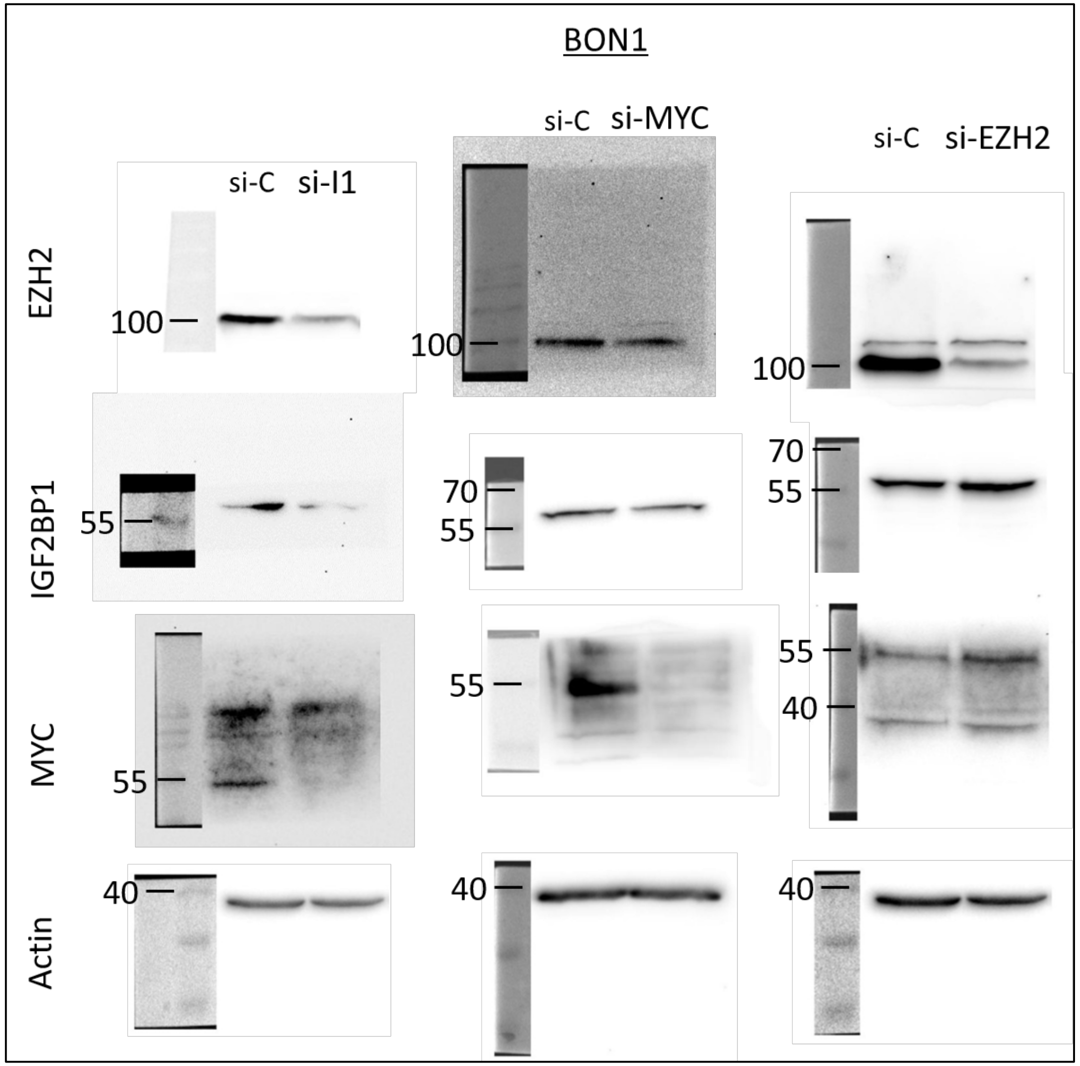

Figure 5G. Western blot of PARP, MYC, IGF2BP1, EZH2, H3K27m3 andH3 in BON1 cells after treatment with BTYBN, DZNep and OTX015 after 72h incomparison to mock and DMSO control cells. Actin served as loading control. Leftmost or rightmost lanes were loaded with PageRuler™ Prestained Protein Ladder. Molecular weights are in KDa units.

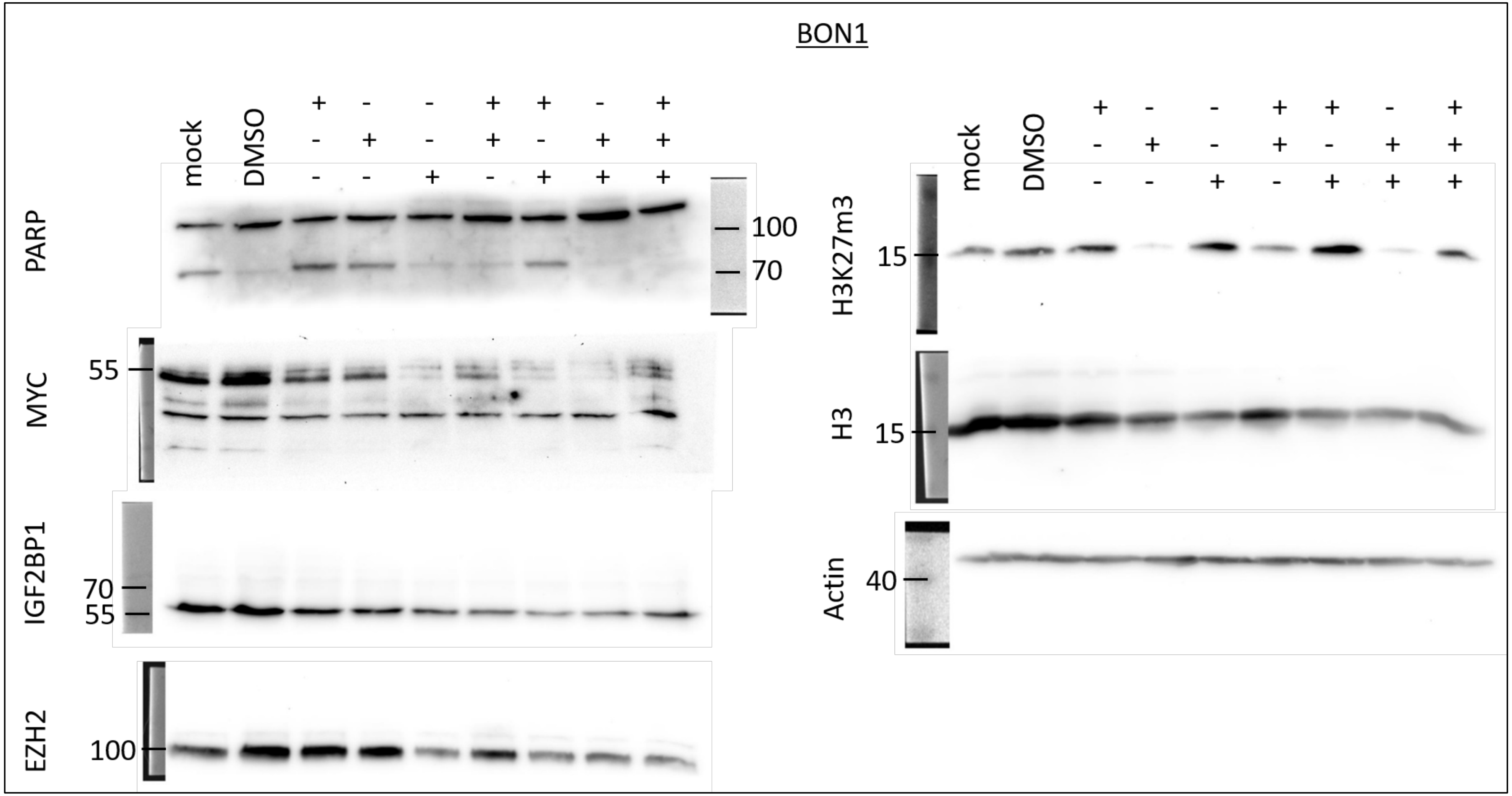

Figure S4A. Western blot of IGF2BP1 in the pulldown lysates of RNA-Immunoprecipitation (RIP) compared to IgG control in BON1, Colo320 and NCIH727 cell lines. Actin served as loading control. Leftmost lanes were loaded with PageRuler™ Prestained Protein Ladder. Molecular weights are in KDa units.

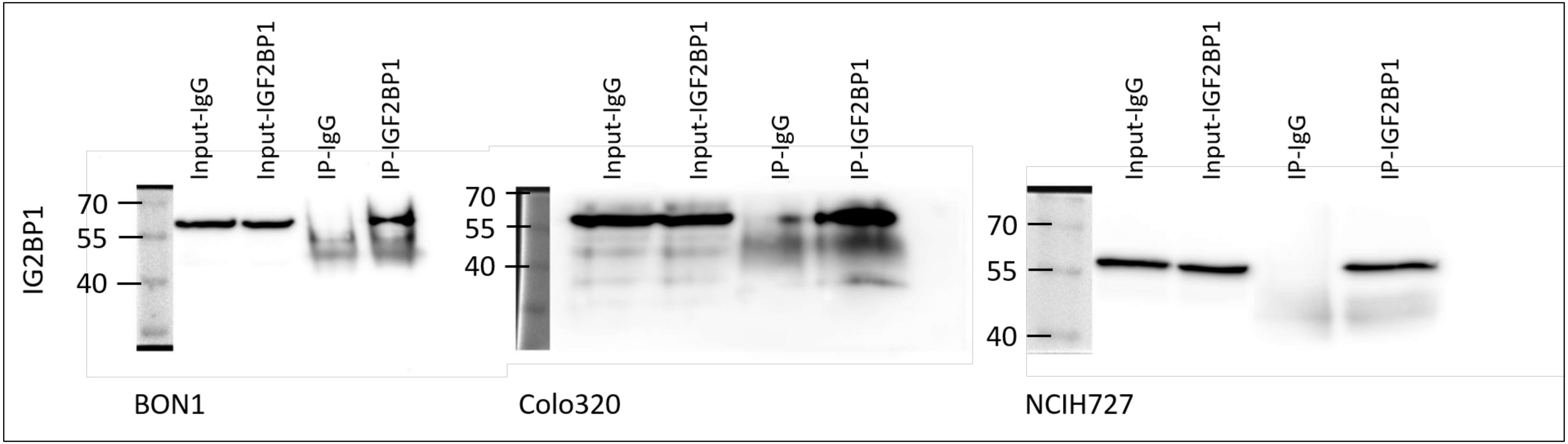

Figure S4C. Western blot of SUZ12 protein 72h after siRNA transfection of METTL3/14 . Actin served as loading control. Leftmost lanes were loaded with PageRuler™ Prestained Protein Ladder. Molecular weights are in KDa units.

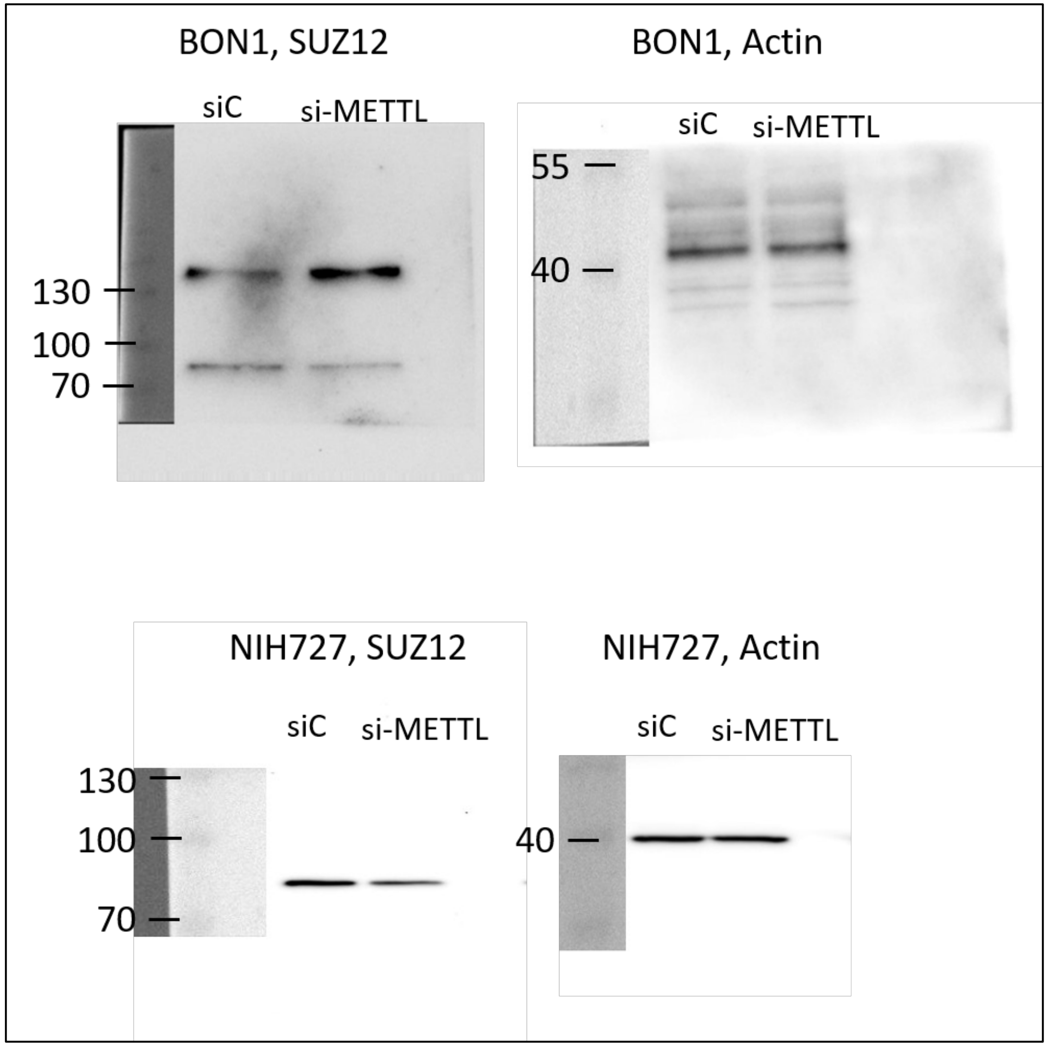

Figure S4D. Western blot of SUZ12 and its potential targets H3K27me3, H3 and p27 in NEN cells after IGF2BP1 knockdown. Actin served as loading control. Leftmost lanes were loaded with PageRuler™ Prestained Protein Ladder. Molecular weights are in KDa units.

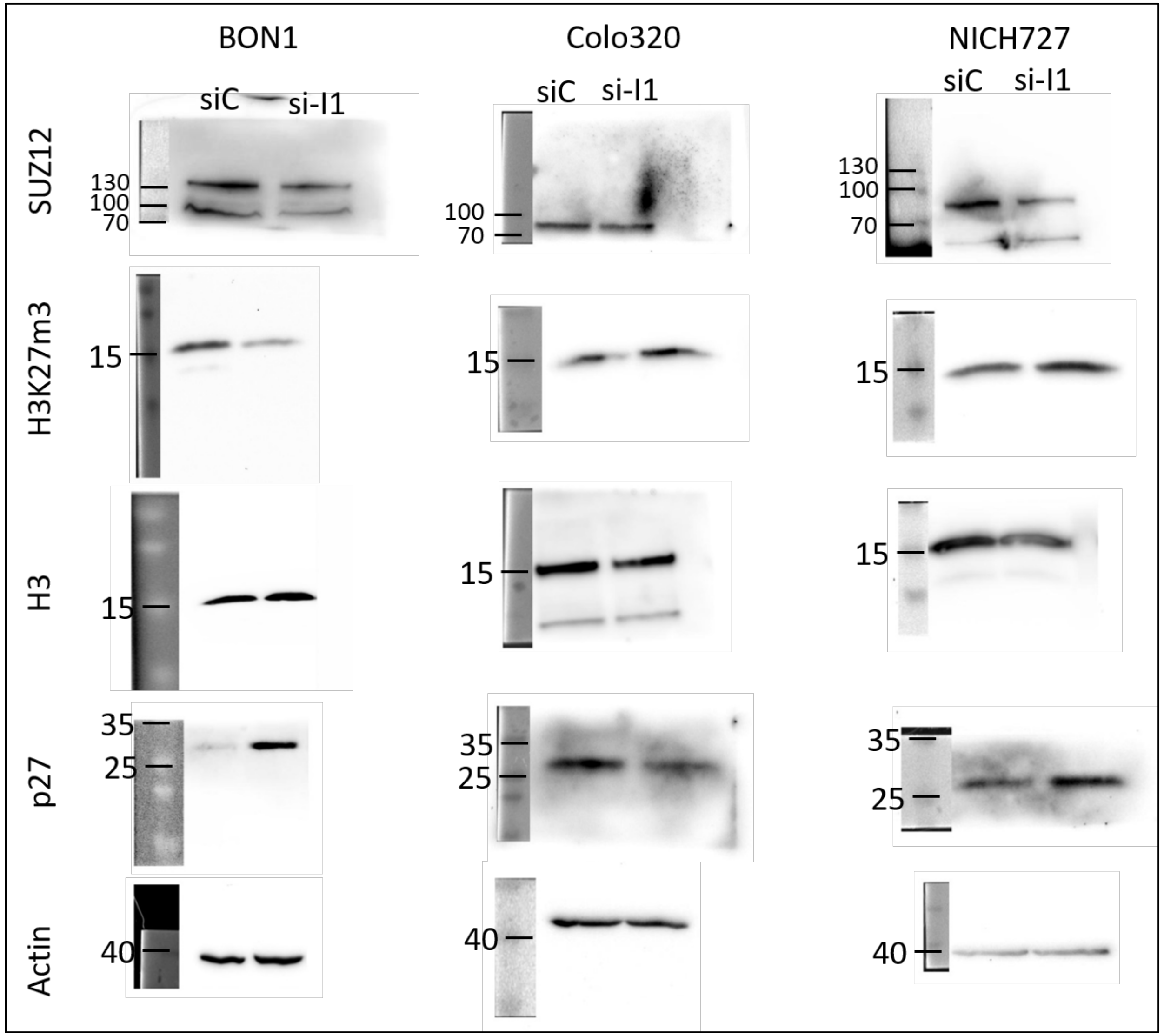

Supplementary Figure Legends

Figure S1. IGF2BP1 expression in NEN cell lines. **(A)** Description of NEN-cell lines (left panel) used in this study and mRNA expression of IGF2BP1 (right panel) analyzed by qRT-PCR normalized to ribosomal protein RPLP0 as internal standard. **(B)** IGF2BP1 mRNA levels in NEN cell lines 72h post transfection with IGF2BP1 siRNA pool (siI1) normalized to ribosomal protein RPLP0 as internal standard and shown as fold change to control transfected cells (siC). **(C)** Cell proliferation was determined by cell counting at indicated time points in NEN cell lines upon knockdown of IGF2BP1 compared to control (siC) transfected cells. **(D)** Cell cycle phase analysis after 72h of IGF2BP1 knockdown in NEN cells. Statistical significance was determined by Student's *t*-test: * $p < 0.05$; ** $p < 0.01$; *** $p < 0.001$.

Figure S2. IGF2BP1 knockdown and apoptosis in NEN cell lines. (A) Representative flow cytometry analysis of apoptosis in the indicated NEN cell lines. Apoptosis was determined by Annexin V / Propidium iodide -staining in NEN cell lines upon knockdown of IGF2BP1 (si-I1) compared to control (siC) transfected cells after 72h of IGF2BP1 knockdown. The four squares (Q1-Q4) indicate how many cells are vital (Q4) or in the early (Q3) or late (Q2) state of apoptosis. The numbers in the squares represent the percentage of positive cells.

Figure S3. IGF2BP1 knockdown affects PRC2 complex and network. **(A)** KEGG-pathway analysis of upregulated mRNAs (threshold: FDR ≤ 0.01) upon IGF2BP1 knockdown in BON1 cells. **(B)** Schematic structure of PRC2 complex (left panel) and regulation of PRC2 associated members from RNA-seq experiment of si-IGF2BP1 compared to si-C transfected BON1 cells. **(C)** Validation of potential IGF2BP1 and EZH2 regulated target mRNAs lncRNAs by qRT-PCR analysis upon IGF2BP1 knockdown (72h) in NEN cell lines. Expression was normalized to ribosomal protein RPLP0 as internal standard and shown as fold change to siC which was set to one. Statistical significance was determined by Student's t-test: (blue) downregulation * $p < 0.05$, (red) upregulation ** $p < 0.05$, ns (grey).

Figure S4. IGF2BP1 regulates stability of PRC2 components. **(A)** Representative western blot of IGF2BP1 in the pulldown lysates of RNA-Immunoprecipitation (RIP) compared to IgG control in BON1, Colo320 and NCIH727 cell lines. **(B)** RIP analysis by qRT-PCR after pulldown of IGF2BP1 protein. Binding of MYC and EZH2 mRNA to IGF2BP1 was compared to IgG isotype control. TERT mRNA served as negative control and showed no accumulation. All amplifications were normalized to total RNA Input. Graphs represent the mean ± SD of three independent experiments. **(C)** Schematic mechanism of m6A-modification of mRNAs by METTL3/14 as „writers" and IGF2BP1 as „reader" (left panel). Regulation of SUZ12 mRNA and the lncRNA HOTAIR in BON1 and NCIH727 cells after METTL3/14 knockdown (middle). Representative western blot of SUZ12 protein 72h after siRNA transfection of METTL3/14 (right). Actine served as loading control. **(D)** Representatvive western blot of SUZ12 and its potential targets in NEN cells after IGF2BP1 knockdown. Actin served as loading control. Statistical significance was determined by Student's *t*-test: * $p < 0.05$; ns, not significant.

Figure S5. Pharmacological inhibition of IGF2BP1-MYC-EZH2 network. **(A)** Phenotypic analysis of Colo320 cells 72h upon indicated treatment strategies. Cell viability was determined by ATP-based CellTiter Glo assay after 72 hours of treatment (left panel) and normalized to DMSO treated control cells. Expression value in the control group was set to 1. Proliferation was analyzed by cell counting at indicated time points (middle). Cell cycle analysis by flow cytometry (right panel) showing increased G1-and sub-G1 phases after pharmacological treatment compared to DMSO control cells. Sub-G1 (black), G1 (light grey), S (grey) and G2 (dark grey) phase. **(B)** Annexin V staining of NEN cell lines showing an increase of apoptotic cells after treatment compared to DMSO treated control cells. Apoptotic cells were calculated as the sum of the individual Annexin V and Propidium iodide cells as well as the double positive cells. All experiments were performed for three times and the data present as mean ± SD. Statistical significance was determined by Student's *t*-test: * $p < 0.05$; ** $p < 0.01$; *** $p < 0.001$.

Figure S6. Pharmacological inhibition of IGF2BP1-MYC-EZH2 network on apoptosis. **(A)** Representative flow cytometry analysis of apoptosis in the indicated NEN cell lines after 72 hours of treatments (header) in comparison to DMSO treated control cells. Apoptosis was determined by Annexin V / Propidium iodide –staining. The four squares (Q1-Q4) indicate how many cells are vital (Q4) or in the early (Q3) or late (Q2) state of apoptosis. The numbers in the squares represent the percentage of positive cells. BT-DZ-OTX: BTYNB-DZNep-OTX015 combination.

Figure S7. Uncropped Western Blot images used in this study.
